# Cortico‐Striatal‐Midbrain Circuit Dysregulation Underlying MK‐801 Induced Impulsivity and the Ameliorative Effects of SEP

**DOI:** 10.1002/advs.202502079

**Published:** 2025-10-24

**Authors:** Xueru Wang, Qinyu Li, Zijie Li, Xuejiao Wang, Hui Yuan, Pingting Yang, Ling Qin

**Affiliations:** ^1^ Laboratory of Hearing Research, School of Life Sciences China Medical University Shenyang Liaoning 110122 China; ^2^ Department of Physiology China Medical University Shenyang Liaoning 110122 China; ^3^ Department of Rheumatology and Immunology The First Hospital of China Medical University Shenyang 110001 China

**Keywords:** dopamine, impulsivity, NMDAR antagonists, schizophrenia, trace amine‐associated receptor

## Abstract

Impulsivity is a core pathological feature of various psychiatric disorders including obsessive‐compulsive disorder, attention‐deficit/hyperactivity disorder, and schizophrenia, which is conceptualized as dysregulated motivational and inhibitory processes. However, the underlying neural mechanism is poorly known. Using an olfactory cued Go/No‐Go task, this work finds increased false alarm rate, shortened licking onset latency, elevated licking frequency and reduced inter‐trial consistency in mice treated by the NMDAR antagonist (MK‐801). Fiber optic recordings reveal MK‐801 enhanced cue‐evoked neuronal responses in the olfactory bulb (OB), orbitofrontal cortex (OFC), ventral tegmental area (VTA), and nucleus accumbens (NAc), while reducing reward‐predictive cue selectivity and increasing NAc dopamine release. Additionally, in vivo electrophysiological recordings demonstrate that theta oscillation power in the NAc and its coherence with OB, OFC, and VTA are also amplified. MK‐801 treatment enhances the correlation between firing rates of NAc neurons and cue, decreases the association with reward, strengthens the correlation with licking and increases the probability of firing peak preceding lick onset. Furthermore, this work confirms that the application of the trace amine‐associated receptor 1 agonist SEP‐363856 could correct the abnormal responses of NAc neurons and behavioral deficits. These findings elucidate the neural basis of impulsivity and support the potential therapeutic effect of SEP.

## Graphical abstract

Impulsivity is a symptom across multiple psychiatric disorders, yet its neural basis remains elusive. This study shows that the NMDAR antagonist MK‐801 elevates dopamine release and disrupts neuronal selectivity in the nucleus accumbens, leading to impulsive behavior. Notably, treatment with SEP‐363856 successfully corrected both neural and behavioral abnormalities.

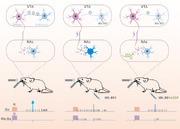

## 1 Introduction

Impulsivity is a tendency toward rapid, unplanned actions with inadequate regard for potential negative consequences.^[^ ^1^ ^]^ It represents a core pathological feature across various psychiatric disorders, including obsessive‐compulsive disorder (OCD), attention‐deficit/hyperactivity disorder (ADHD), and schizophrenia (SZ).^[^ ^2^, ^3^, ^4^ ^]^ Elevated impulsivity is associated with several maladaptive behaviors, such as substance abuse, increased suicide risk, and criminality.^[^ ^1^, ^5^ ^]^ Despite its clinical significance, the pathophysiological mechanisms underlying impulsivity remain poorly understood due to a lack of in vivo neurophysiological and pharmacological research.

Impulsivity is typically operationalized as the tendency to generate premature responses due to dysregulated motivational and inhibitory processes. Laboratory paradigms such as the Go/No‐Go task provide objective measures of impulsivity,^[^ ^6^ ^]^ in which subjects must respond to a “Go” stimulus but withhold responses to a “No‐Go” stimulus. Individuals with high impulsivity typically exhibit increased false alarm (FA) rates and shorter reaction times.^[^ ^7^, ^8^ ^]^ This behavioral impairment is associated with deficits in higher‐order cognitive processes, including executive function, reward processing, and motor control.^[^ ^9^ ^]^ At the neural level, impulsivity has been linked to dysfunctions within cortical‐striatal circuits,^[^ ^10^ ^]^ which integrate motivation with action selection and control. Neuroimaging studies have found that the blood oxygen level dependent response signals in the striatum and orbitofrontal cortex (OFC) are enhanced under the “No‐Go” condition.^[^ ^11^ ^]^ Dysfunctional activities in striatum and OFC were also observed in OCD characterized with impulsivity.^[^ ^12^, ^13^ ^]^ Additionally, modulation of nucleus accumbens (NAc) activity through deep brain stimulation has been shown to restore the impulsive behaviors in OCD, supporting the critical role for cortico‐striatal circuits.^[^ ^14^ ^]^ Dopaminergic (DAergic) activity is a critical modulator of cortico‐striatal function, shaping both motivational drive and response control.^[^ ^10^ ^]^ DA released from the ventral tegmental area (VTA) into the striatum plays a key role in response control, facilitating movement execution and inhibiting inappropriate behaviors.^[^ ^15^, ^16^ ^]^ Optogenetic activation of VTA DAergic neurons and the VTA‐NAc pathway has been shown to increase impulsivity.^[^ ^17^, ^18^ ^]^ These findings suggested that DA dysregulation in VTA‐NAc pathway plays a key role in impulsive behaviors, although the underlying mechanisms remain unclear.

To address this question, combined behavioral and neurophysiological investigations in suitable animal models are necessary. Mice administered by N‐methyl‐D‐aspartate receptor (NMDAR) antagonists, such as MK‐801, are well utilized as animal models for SZ‐like behavioral abnormalities.^[^ ^19^, ^20^ ^]^ Impulsivity is strongly associated with positive symptoms and cognitive dysfunction in SZ,^[^ ^1^, ^5^ ^]^ and elevated striatal DA levels^[^ ^21^, ^22^ ^]^ and receptor density have been consistently shown in SZ patients.^[^ ^23^ ^]^ However, the specific neural mechanisms linking DA abnormalities to impulsivity in MK‐801 treated model remain unclear. Therefore, in this study, we aim to elucidate this issue.

Given the high olfactory sensitivity of mice and their widespread use in behavioral experiments,^[^ ^24^ ^]^ we used the olfactory‐cued Go/No‐Go behavioral paradigm in this study. The choice of an olfactory task is also supported by the close anatomical and functional association between olfactory circuitry and brain regions governing motivation and action control, which were frequently impaired in SZ.^[^ ^25^, ^26^, ^27^ ^]^ For this, we employed an odor discrimination task (ODT) within Go/No‐Go paradigm to assess impulsivity in MK‐801‐induced SZ model mice, and used fiber photometry to monitor calcium activity in glutamatergic and GABAergic neurons in the olfactory bulb (OB), OFC, VTA, and NAc. Dynamic DA levels in the NAc were measured using the DA4.4 biosensor, and in vivo electrophysiological recordings were conducted to evaluate the oscillations of local field potential (LFP) and single unit activity (SUA).

Additionally, we investigated the therapeutic potential of SEP‐363856 (SEP), a trace amine‐associated receptor 1 (TAAR1) agonist in Phase III clinical trials for SZ.^[^ ^28^ ^]^ Preclinical studies suggest that TAAR1 activation normalizes hyperdopaminergic activity by reducing DA synthesis and release.^[^ ^29^ ^]^ SEP has shown promise in improving SZ‐like behaviors, including hyperactivity and sensorimotor gating deficits, in animal models.^[^ ^28^ ^]^ Furthermore, activation of TAAR1 can also enhance the ability for response control in both primates and rodents.^[^ ^30^, ^31^ ^]^ Here, we validated effects of SEP on MK‐801‐induced impulsivity, and abnormalities of DA release and neural activity.

## 2 Results

### 2.1 MK‐801 Treatment Altered Response Patterns in Mice Performing the ODT

**Figure 1:**
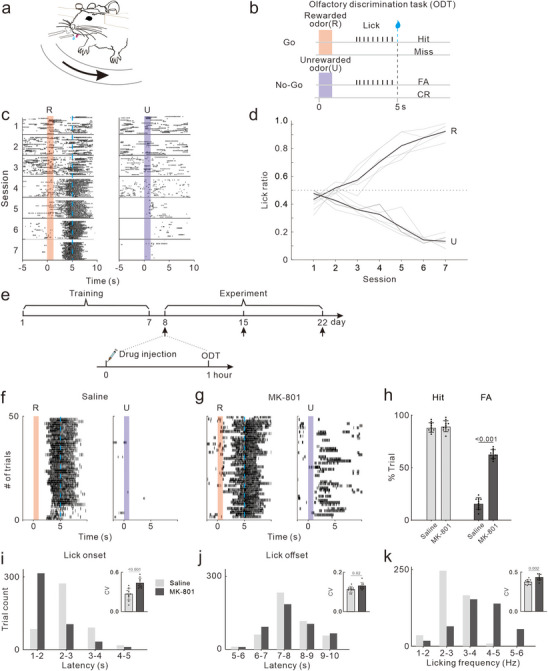
Effects of MK‐801 treatment on behavioral performance in ODT. a,b) Schematic of the behavioral setup and the task structure. Head‐fixed mice were presented with Rewarded (R) or Unrewarded odor (U). Only odor R was paired with a water reward if an animal licked after odor stimulus. c) Example lick plot from an individual mouse performing the task across 7 sessions. Lick responses to odors are shown for individual trials during each session, sorted in columns according to odor, with early trials at the top and late trials at the bottom for each session. Black tick marks, individual lick contacts with the spout; red/blue bars, stimulus presentation. d) Function of lick ratio against training session. Gray, result of individual mouse; black, averaged across the mice; dotted line, a ratio score of 0.5, which indicates random performance. e) The experimental timeline for training, drug treatments and behavioral tests. f,g) Representative raster plots of licking behavior recorded from one mouse after Saline (f) and MK‐801(g) treatments. h) Statistical charts of Hit rate (light gray bars) and FA rate (dark gray bars). Dots represent the values of individual mouse. Bars and ticks are means ± SD, Student's *t*‐test. i) Frequency distribution of lick onset latencies.  Insert: CV of onset latency. j,k) Same as (i), but for lick offset latency and licking frequency.

Mice are trained to discriminate between two odors in a Go/No‐Go task, assessing their capacity to react correctly to reward‐associated olfactory cues. As shown in **Figure** **1**a,b, in Go trials, mice receive a water reward for licking the spout after a rewarded odor (R) is presented (0–1s, Hit); failing to lick results in no reward (Miss). Conversely, in No‐Go trials, licking in response to an unrewarded odor (U) is counted as a false alarm (FA), while no licking is a correct rejection (CR).

The performance of mice during the training process is illustrated in Figure 1c. Initially, during the first three training sessions, the mice exhibited random licking behaviors, which were not contingent upon the timing or type of odor stimulus. As training progressed, the licking gradually became concentrated around the time point of reward delivery, with a reduction in licking during non‐reward periods. Ultimately, the licking actions predominantly concentrated between the end of the odor stimulation and the onset of the reward, indicating that the mice had established an association between the conditioned stimulus (odor R) and the unconditioned stimulus (water reward). Figure 1d illustrates the evolution of licking ratios among 5 mice in response to odor stimulus, highlighting an increase in discrimination with training. With about 1 week of training, mice were able to accurately discriminate between the two distinct odor stimuli (correct rate ≥ 0.8). The correct rate is calculated using the formula: correct rate = (Hit rate + (1 – FA rate)) / 2.

Once the mice achieved a correct rate of 0.8 or higher for three consecutive sessions, we proceeded with the in vivo pharmacological intervention experiments. We first assessed the behavioral dose–response profile of MK‐801 and evaluated the persistence of its effects across time (Figure S1a,b, Supporting Information). Based on these results, we selected a dose of 0.2 mg kg^−1^ and the earliest time point of 1 h post‐injection, which produced reliable behavioral alterations, for subsequent experiments. Each mouse underwent one experiment per week with daily maintenance training. The order in which each mouse received the drug injections was randomly assigned (Figure 1e). As depicted in Figure 1f, the odor preference remained unchanged following saline treatment, with lick responses being triggered by the termination of odor R stimulation. But after MK‐801 treatment (Figure 1g), the mice exhibited persistent lick responses to odor R while also initiating responses to odor U, suggesting a failure to inhibit inappropriate lick responses in compliance with established protocols.

In our analysis of 10 mice across different experimental conditions, we assessed the Hit rate (the ratio of hit trials to go trials) and the FA rate (the ratio of FA trials to no‐go trials). We found that MK‐801 treatment significantly increased the FA rate in mice (t_18_ = 21.21, *p <* 0.001), but had no significant effect on the Hit rate (t_18_ = 0.55, *p =* 0.59; Figure 1h). We further characterized the frequency distributions of lick onset and offset latencies (Figure 1i,j). After saline injection, mice exhibited lick onsets predominantly within the 2–3 s after cue presentation. But MK‐801 treatment significantly shortened the onset latency, with most licking responses occurring between 1 and 2 s. Conversely, the distributions of lick offset latencies were similar between groups, both peaking ≈7–8 s after cue presentation. We further assessed trial‐to‐trial consistency of lick latency and found that the coefficient of variation (CV) was markedly increased following MK‐801 treatment (t_18_ = 4.60, *p <* 0.001, Figure 1i; t_18_ = 2.55, *p =* 0.02, Figure 1j). The frequency of licking (0 – 5 s after cue onset) and its CV were significantly increased by MK‐801 treatment (t_18_ = 3.64, *p =* 0.002, Figure 1k). These results demonstrate that MK‐801 leads to prematurely initiated and more variable licking responses. To assess the effects of MK‐801 on general motor activity, we also performed open field test (OFT) after 1 h post‐injection. Compared to saline controls, MK‐801 treatment significantly increased total distance traveled (t_18_ = 9.12, *p <* 0.001, Figure S1b, Supporting Information). Thus, anarchic licking behavior and hyperactivity co‐occurred after MK‐801 treatment.

### 2.2 Neural Calcium Activity Abnormalities in the OB During ODT in MK‐801‐Induced SZ Model Mice

**Figure 2:**
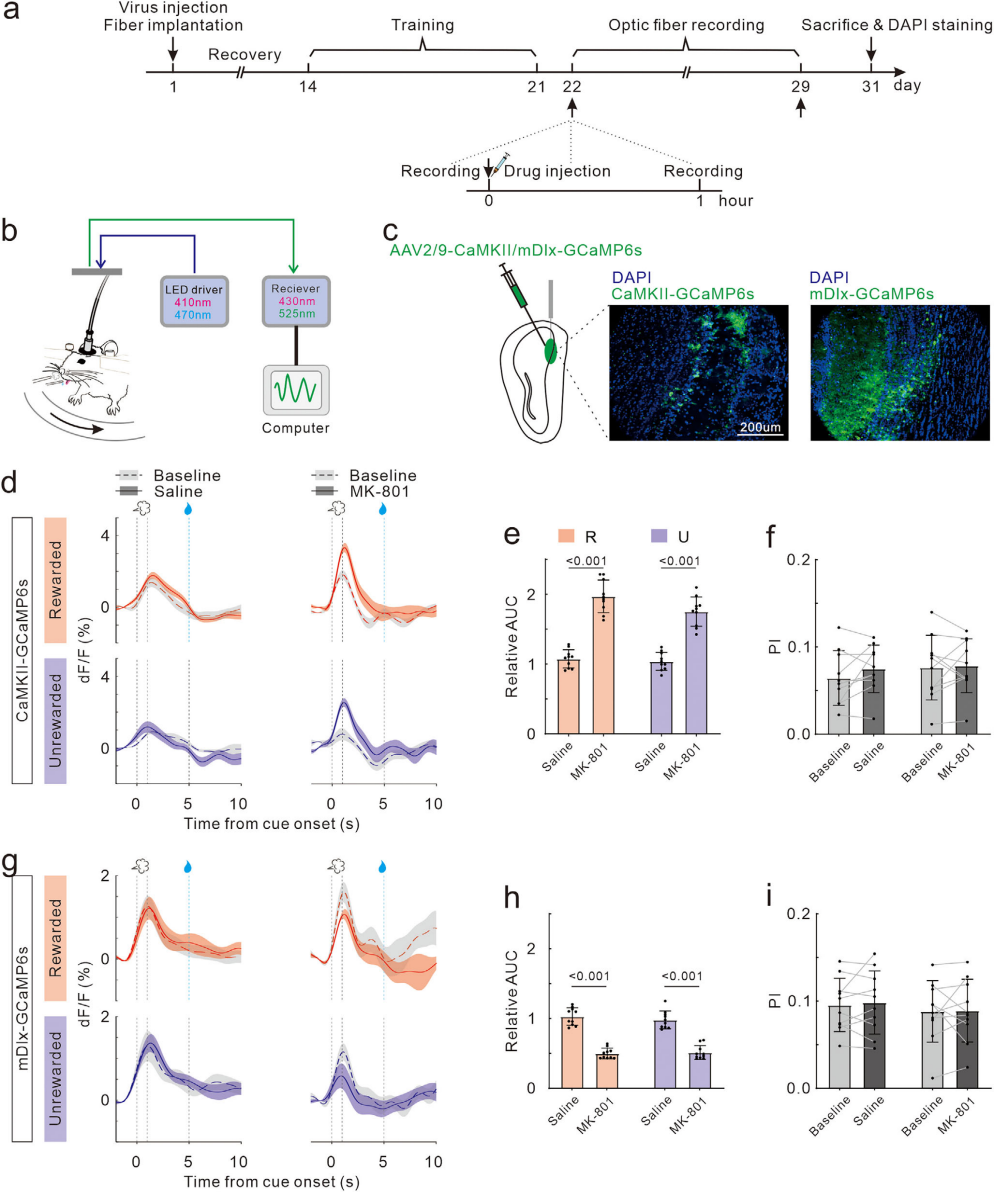
Effects of MK‐801 treatment on neural calcium activity in OB during ODT. a) Timeline of virus injection, training, drug injections, optic fiber recording and DAPI staining. b) Schematic demonstrating the path of excitation LEDs and reporter emission signals during fiber photometry recording. c) Left: diagram showing targeting of AAV injection and fiber implantation to the OB. Right: representative coronal section images displaying CaMKII‐GCaMP6s and mDlx‐GCaMP6s expressions (green, GCaMP fluorescence; blue, DAPI nuclear counterstain; scale bar: 200 µm). d) Trial‐averaged traces (mean ± SE) of the calcium responses evoked by Rewarded odor (top) and Unrewarded odor (bottom) in glutamatergic neurons of OB from a representative mouse. Baseline responses are indicated by dotted lines with gray shading. Post‐treatment responses are represented by solid lines with colored shading: Saline (left panel) and MK‐801 (right panel). e) Statistical charts of relative AUC in R trials (red bars) and U trails (blue bars) for glutamatergic neurons. Dots represent the values of individual mouse. Bars and ticks are means ± SD, Student's *t*‐test. f) Comparison of DI between baseline and post‐treatment in glutamatergic neurons of two groups: Saline and MK‐801 treated. Paired Student's *t*‐test. g) Same as (d), but for GABAergic neurons of OB. h) Same as (e), but for GABAergic neurons of OB. i) Same as (f), but for GABAergic neurons of OB.

To further investigate the neural activities during ODT, we used fiber photometry to record the immediate calcium signals from glutamatergic and GABAergic neurons in OB (**Figure** **2**a). We injected rAAV‐CaMKIIa‐GCaMP6 or rAAV‐mDlx‐GCaMP6 into OB, respectively.

Mice initiated ODT training 2 weeks post the optical fiber implantation surgery. Upon task mastery, fiber optic recordings were taken while the mice performed the task (consisting of 10 Go trials and 10 No‐Go trials in a pseudo‐randomized sequence). The recordings were performed at pre‐injection (baseline) and 1h post‐injection of either saline or MK‐801. After a 7‐day washout period, the drug was changed, and the fiber optic recordings were repeated. The order of injection for saline or MK‐801 was randomized. Each mouse maintained daily training when not undergoing fiber optic recording (Figure 2a,b). 1 day after the final recording, perfusion and brain tissue collection were conducted, and only the data with correct placement of fiber implantation was included in the analysis (Figure 2c).

The fluorescence traces (mean ± SE) in Figure 2d depicted examples of temporal calcium fluctuations upon odor stimulus in glutamatergic neurons of OB within one session. At baseline, the calcium signals of OB glutamatergic neurons were significantly increased (calcium transients) in response to odor R and U. Saline treatment did not alter the transient responses. However, MK‐801 treatment significantly amplified the magnitude of calcium transients triggered by both odor R and U. The similar calcium transients evoked by odor R and U were observed in OB GABAergic neurons at baseline. The transient responses were unaffected by saline treatment but were substantially reduced following MK‐801 treatment (Figure 2g).

We quantified the area under the curve (AUC) of odor‐evoked calcium transient peaks in 10 mice, and calculated the relative AUC as the ratio of post‐to‐pre drug administration. Our results indicated that saline treatment maintained the AUC ratio for glutamatergic neuronal calcium transients near unity, while MK‐801 treatment doubled it (t_18_ = 10.61, *p <* 0.001; t_18_ = 9.19, *p <* 0.001; Figure 2e). Conversely, the AUC ratio for GABAergic neurons significantly decreased post‐MK‐801 treatment (t_18_ = 11.43, *p <* 0.001; t_18_ = 9.13, *p <* 0.001; Figure 2h). Additionally, we computed the discrimination index (DI) to evaluate the selectivity of neuronal responses to odor R and U, utilizing the formula DI = |(AUC_R –_ AUC_U_)| / (AUC_R_ + AUC_U_). At baseline, the DI for both glutamatergic and GABAergic neuronal calcium transients in OB were low. Neither saline nor MK‐801 treatment elevated these values (t_9_ = 1.43, *p =* 0.18; t_9_ = 0.29, *p =* 0.18; Figure 2f; and t_9_ = 0.47, *p =* 0.65; t_9_ = 0.09, *p =* 0.93; Figure 2i).

### 2.3 Neural Calcium Activity Abnormalities in the OFC During ODT in MK‐801‐Induced SZ Model Mice

**Figure 3:**
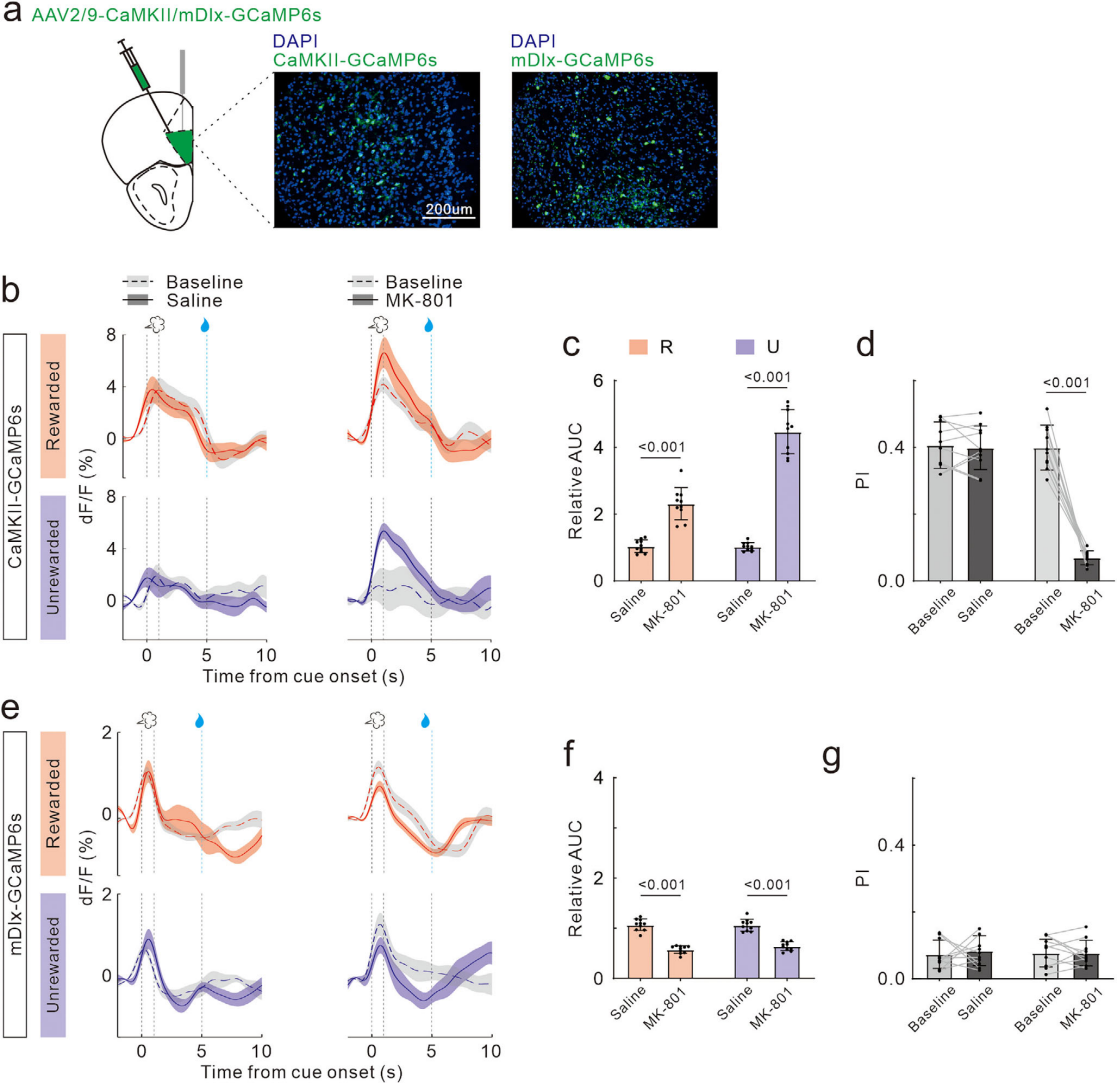
Effects of MK‐801 treatment on neural calcium activity in OFC during ODT. Same format as for Figure 2c‐i.

In a parallel approach, we injected rAAV‐CaMKIIa‐GCaMP6 or rAAV‐mDlx‐GCaMP6 into the OFC to monitor the calcium activity of glutamatergic and GABAergic neurons during ODT (**Figure** **3**a). At baseline, OFC glutamatergic neurons showed a significant increase in calcium signals following odor R delivery, with a subtler response to odor U. The response profile remained unchanged with saline treatment. However, MK‐801 treatment significantly increased the magnitude of calcium transients evoked by both odor R and U (Figure 3b). GABAergic neurons in OFC demonstrated increases in calcium signals in response to both odor R and U. The transient responses remained stable following saline treatment but were significantly reduced by MK‐801 injection (Figure 3e). Through quantitative analysis of the AUC ratio and DI, we further confirmed that MK‐801 treatment significantly enhanced the response intensity of OFC glutamatergic neurons to odor stimulus (t_18_ = 7.81, *p <* 0.001; t_18_ = 16.16, *p <* 0.001; Figure 3c) and diminished their odor selectivity (t_9_ = 0.43, *p =* 0.68; t_9_ = 12.28, *p <* 0.001; Figure 3d). Moreover, MK‐801 reduced the response intensity of GABAergic neurons (t_18_ = 11.35, *p <* 0.001; t_18_ = 8.98, *p <* 0.001; Figure 3f), but had no effect on the selectivity (t_9_ = 0.17, *p =* 0.87; t_9_ = 0.51, *p =* 0.62; Figure 3g).

### 2.4 Neural Calcium Activity Abnormalities in the VTA During ODT in MK‐801‐Induced SZ Model Mice

**Figure 4:**
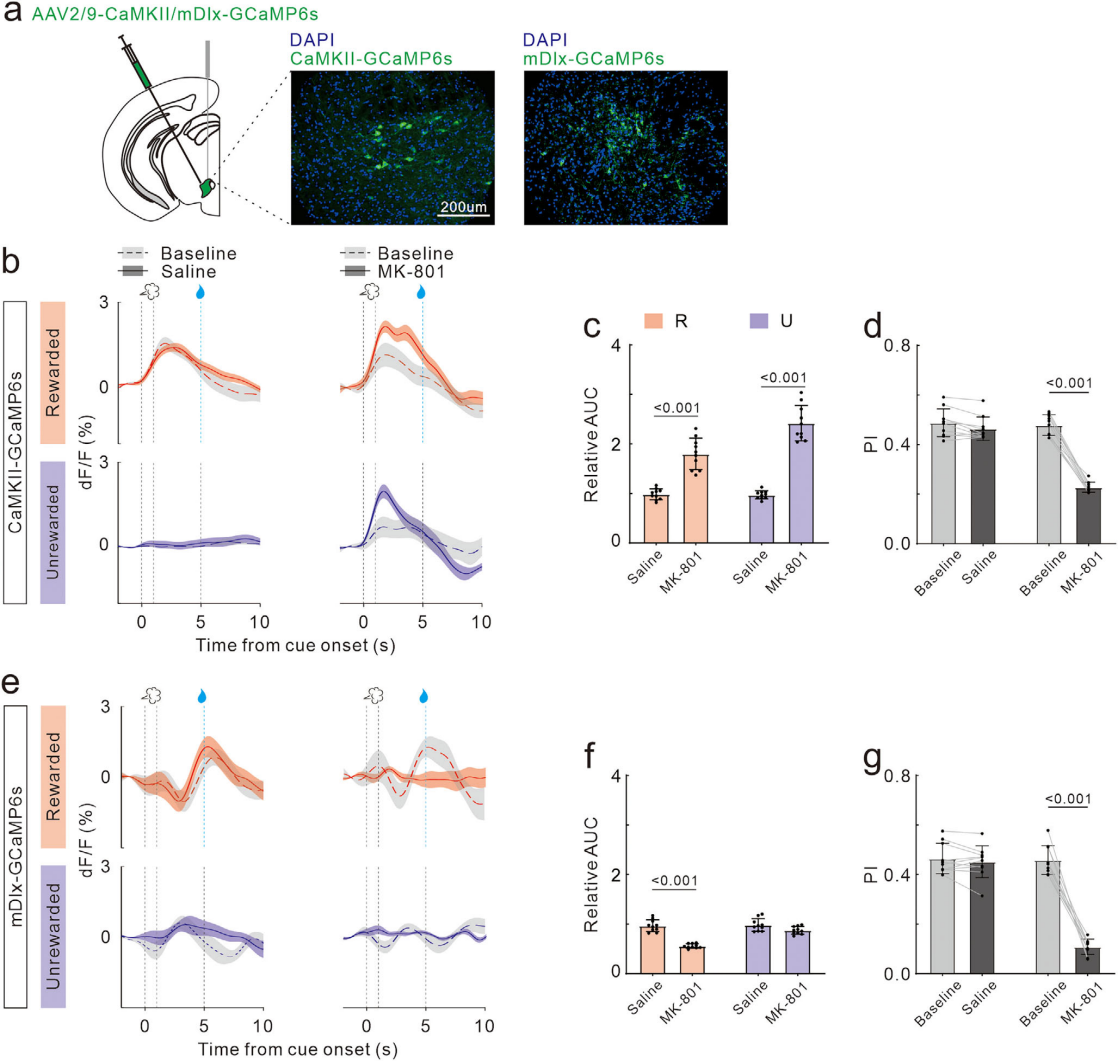
Effects of MK‐801 treatment on neural calcium activity in VTA during ODT. Same format as for Figure 2c‐i.

We recorded calcium signals from glutamatergic and GABAergic neurons in the VTA, respectively (**Figure** **4**a). At baseline, VTA glutamatergic neurons exhibited a transient response to odor stimulus onset in R trials, returning to base levels before the reward (Figure 4b). GABAergic neurons responded to both the odor stimulus and reward in R trials, with a stronger reaction to the reward (Figure 4e). We hypothesized that the inhibitory effect of GABAergic neurons on glutamatergic neuron activity primarily occurred after the reward, resulting in a strong response to the odor cue and a weak response to the reward in glutamatergic neurons. In U trials, there was no significant response to the odor stimulus.

Saline treatment did not cause any noticeable changes in response properties. However, MK‐801 treatment suppressed the response of GABAergic neurons to both the odor cue and reward, while increasing the response intensity of glutamatergic neurons to odor stimulus, notably to odor U. This resulted in reduced response selectivity and a decline in odor discrimination ability, which may be the cause of the increased FA rate in mice. Statistical analyses of AUC ratio and DI revealed that MK‐801 treatment significantly increased the response intensity of glutamatergic neurons to odor cues (t_18_ = 7.64, *p <* 0.001; t_18_ = 12.55, *p <* 0.001; Figure 4c), diminished that of GABAergic neurons (t_18_ = 10.09, *p <* 0.001; t_18_ = 1.81, *p =* 0.09; Figure 4f), and reduced odor response selectivity in both neuron types (t_9_ = 2.16, *p =* 0.06; t_9_ = 17.14, *p <* 0.001; Figure 4d and t_9_ = 1.07, *p =* 0.31; t_9_ = 13.59, *p <* 0.001; Figure 4g).

### 2.5 Neural Calcium Activity and DA Level Abnormalities in the VTA During ODT in MK‐801‐Induced SZ Model Mice

**Figure 5:**
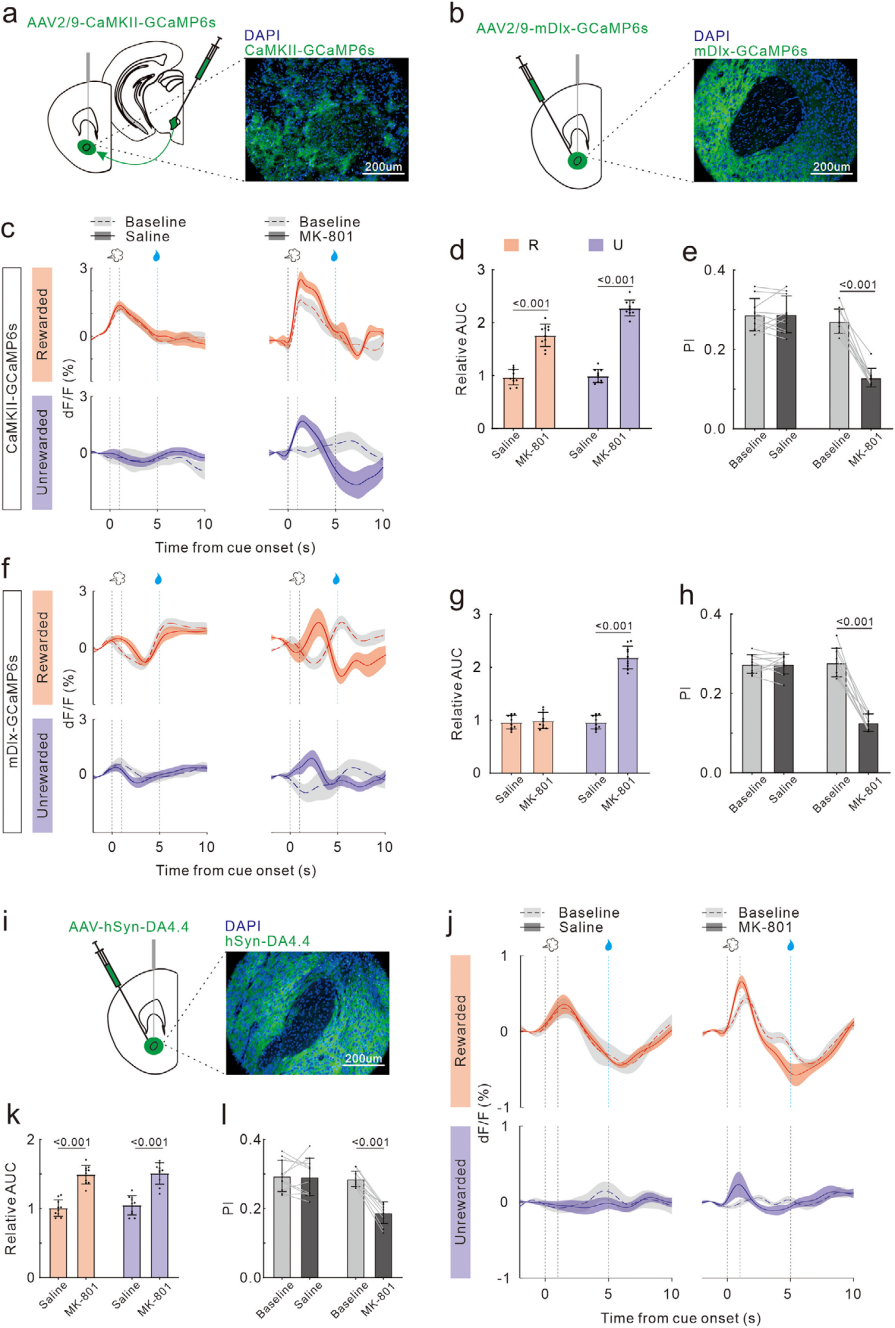
Effects of MK‐801 treatment on neural calcium activity and DA level in NAc during ODT. Same format as for Figure 2c‐i.

Given that the NAc is predominantly composed of GABAergic neurons and receives glutamatergic projections from the VTA,^[^ ^32^, ^33^ ^]^ we targeted the VTA with AAV‐CaMKII‐GCaMP6 virus injections and implanted optical fibers in the NAc to record calcium activity in glutamatergic projections from the VTA to the NAc (**Figure** **5**a). In parallel experiments, we injected AAV‐mDlx‐GCaMP6 virus and implanted fibers into the NAc to record calcium activity in GABAergic neurons (Figure 5b).

Glutamatergic projections in the NAc exhibited a transient response to the odor R stimulus but not to odor U, mirroring the response pattern of VTA glutamatergic neurons (Figure 5c). Similarly, GABAergic neurons in the NAc, akin to those in the VTA, responded to the odor R stimulus and reward, while showing no response to the odor U (Figure 5f). MK‐801 treatment significantly heightened the response intensity of glutamatergic projections to both odor R and U, thereby reducing response selectivity. On the other hand, MK‐801 treatment advanced the peak of calcium transients in GABAergic neurons to precede the reward delivery in R trials and elicited analogous response peaks in U trials.

Statistical analysis further confirmed that MK‐801 significantly increased the response intensity of VTA‐to‐NAc glutamatergic projections to odor stimulus (t_18_ = 9.64, *p <* 0.001; t_18_ = 20.89, *p <* 0.001; Figure 5d) and diminished response selectivity (t_9_ = 0.09, *p =* 0.93; t_9_ = 13.35, *p <* 0.001; Figure 5e). Additionally, it enhanced the response intensity of GABAergic neurons to odor U without affecting responses to odor R (t_18_ = 0.49, *p =* 0.63; t_18_ = 15.44, *p <* 0.001; Figure 5g), leading to a decrease in selectivity (t_9_ = 0.005, *p =* 0.99; t_9_ = 11.04, *p <* 0.001; Figure 5h).

Since the excitability of NAc neurons is modulated by DA from the VTA,^[^ ^34^ ^]^ we injected AAV‐hSyn‐DA4.4 into the NAc and implanted an optical fiber to dynamically monitor DA levels in vivo in mice (Figure 5i). As shown in Figure 5j, under baseline and saline treatment conditions, the fluorescence of DA4.4 transiently increased after odor R but showed no significant change after odor U. MK‐801 treatment led to faster and higher peaks in dF/F evoked by odor R, and odor U also induced peaks. AUC ratio and DI analysis confirmed that MK‐801 significantly increased DA release evoked by odor stimulus (t_18_ = 8.50, *p <* 0.001; t_18_ = 6.99, *p <* 0.001, Figure 5k) and reduced its response selectivity (t_9_ = 0.21, *p =* 0.84; t_9_ = 10.44, *p <* 0.001, Figure 5l). Integrating fiber photometry results, we speculate that MK‐801 inhibited GABAergic neuronal activity in the OB, OFC, and VTA. The inhibition led to enhanced excitatory activity of glutamatergic neurons, resulting in a decreased ability to inhibit lick behavior and an increased FA rate. This may be related to increased DA release from the VTA to the NAc.

### 2.6 MK‐801 Enhanced LFP Theta Oscillations in OB, OFC, VTA, and NAc

**Figure 6:**
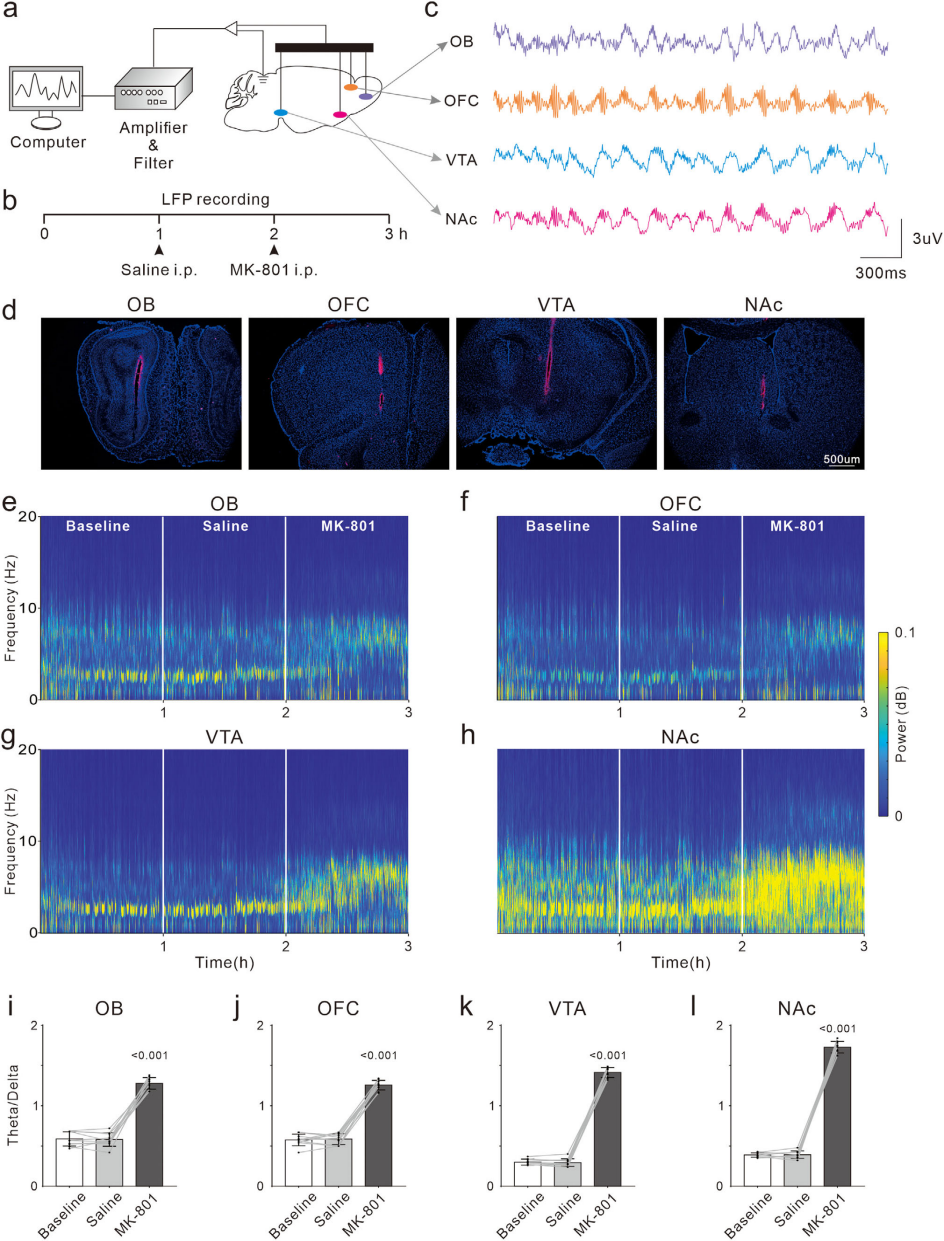
Effects of MK‐801 treatment on LFP oscillations in OB, OFC, VTA and NAc. a) Schematic diagram of electrophysiological recording. b) Timeline of drug treatment and electrophysiological recording. c) Example raw traces of LFPs in the OB, OFC, VTA and NAc. d) Histological images of recording electrode in the OB, OFC, VTA and NAc. e–h) Example temporal‐spectrograms of LFPs in the OB (e), OFC (f), VTA (g) and NAc (h). Two white lines indicate the time points of Saline and MK‐801 treatments, respectively. i–l) Quantification of average Theta/Delta power ratios in the OB (i), OFC (j), VTA (k) and NAc (l) across baseline, Saline‐, and MK‐801 treatment conditions. Dots represent the values of individual recording. Bars and ticks are means ± SD, one‐way repeated measure ANOVA with Tukey's multiple comparisons test.

To assess the effects of MK‐801 on neural activity across multiple brain regions simultaneously, microwire electrodes were implanted in OB, OFC, VTA and NAc of each mouse. We recorded local field potentials (LFPs) in these regions 1 h before and 1 h after the injection of saline and MK‐801 (**Figure** **6**a–c). Representative histological images were presented in Figure 6d, verifying the correct placement of the electrodes in the OB, OFC, VTA, and NAc. The LFP temporal‐spectrogram analysis in Figure 6e–h revealed that during baseline and saline treatment, LFP signal energy was predominantly concentrated in the delta (1–4 Hz) and theta (4–8 Hz) frequency bands, with the delta band power exceeding that of the theta band in the OB, OFC, VTA and NAc. After MK‐801 treatment, the delta band power decreased while theta band power increased. We calculated the theta/delta power ratio from a total of 10 recordings across 5 mice. The results indicated a significant increase in the theta/delta ratio in all regions after MK‐801 treatment, with the most significant difference in NAc (F_(2,9)_ = 309.1, *p <* 0.001, Figure 6i; F_(2,9)_ = 463.1, *p <* 0.001, Figure 6j; F_(2,9)_ = 1954, *p <* 0.001, Figure 6k; F_(2,9)_ = 2413, *p <* 0.001, Figure 6l). To explore the relation between theta power and the impulsive behavior, we further analyzed the LFPs in the NAc during the No‐Go trials of ODT. As shown by the representative LFP power spectra under baseline condition, theta‐band power in FA trials was higher than that in CR trials (Figure S1c, Supporting Information), suggesting that theta power was higher when the behavioral motivation was elevated. Saline injection had no effect on the theta power in FA and CR trials, however, MK‐801treatment significantly increased the theta power.

### 2.7 MK‐801 Enhanced the LFP Coherence between the NAc and Others

**Figure 7:**
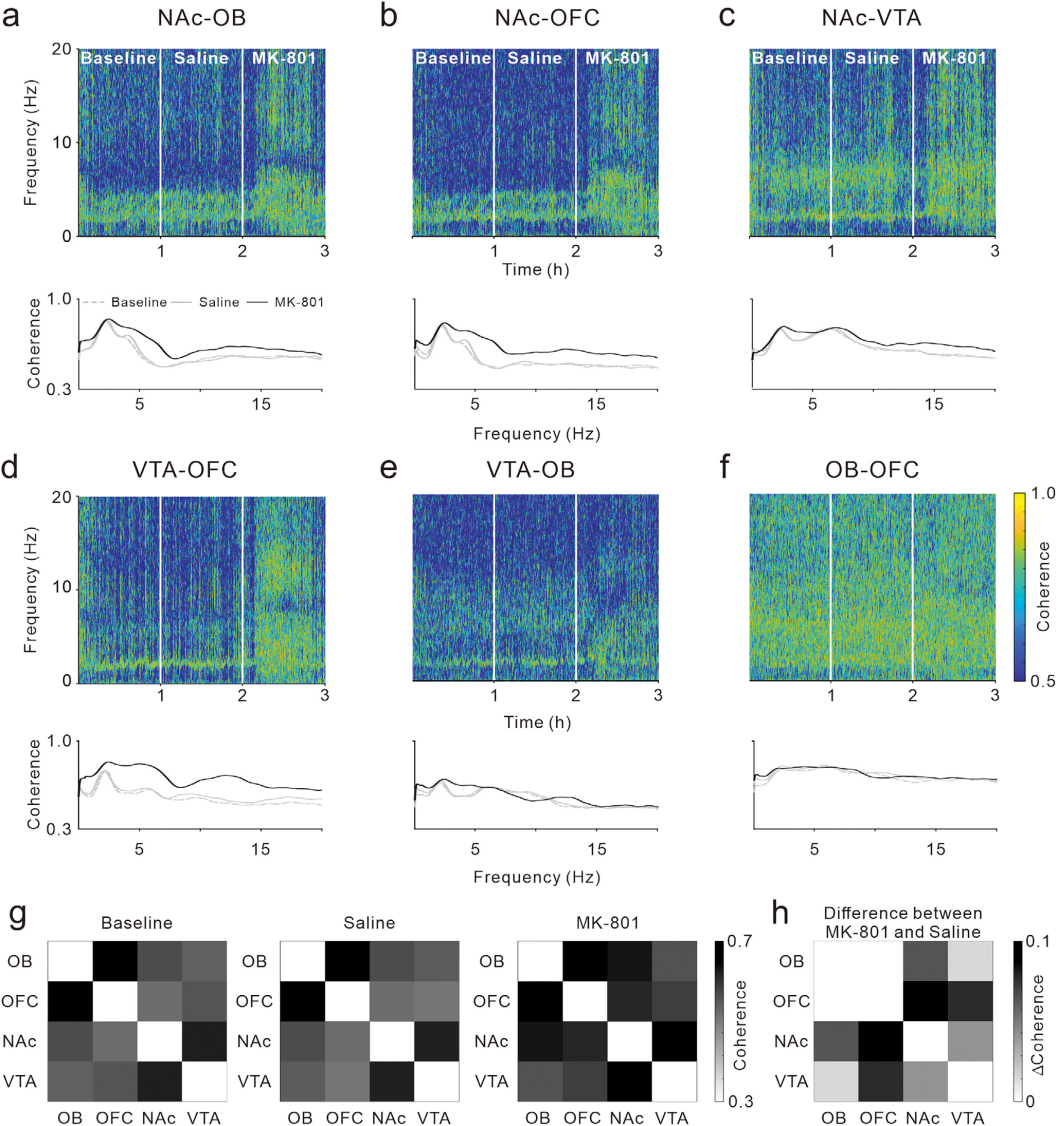
Effects of MK‐801 treatment on LFP coherence between any two recorded brain regions. a–f) Top row displays LFP coherence temporal‐spectrogram between any two recorded brain regions from a representative mouse. Two white lines indicate the time points of Saline and MK‐801 treatments, respectively. Bottom row presents the frequency distribution functions of LFP coherence during three distinct stages: baseline (gray dotted line), after Saline treatment (gray solid line), and after MK‐801 treatment (black solid line). g) Matrices of average LFP coherence across recorded brain regions during three distinct stages: baseline (left), after Saline treatment (middle), and after MK‐801 treatment (right). h) Difference matrix showing the change in LFP coherence between MK‐801 and Saline treatment across brain regions.

To explore the impact of MK‐801 on the synchrony of neuroelectric activity across multiple brain regions, we analyzed the changes in LFP coherence between pairs of brain regions before and after drug treatments. **Figure** **7**a–f presents representative temporal‐spectrograms of LFP coherence (upper panels) and frequency distribution functions (lower panels) from one mouse. The results indicated that at baseline, coherence between the NAc and other regions—NAc‐OB, NAc‐OFC, and NAc‐VTA—had a significant peak in the delta band (a‐c). And NAc‐VTA also showed relatively high coherence in the theta band. Coherence between VTA‐OFC and VTA‐OB also peaked in the delta band, with the VTA‐OB coherence being lower (d and e). Cross‐ structure region communication is notably characterized by delta band activity, while theta band communication predominantly occurs between the NAc and the VTA. The OB‐OFC coherence was robust across all frequency ranges without distinct peak (f). saline treatment did not alter LFP coherences among the brain regions. In contrast, MK‐801 treatment significantly increased coherences within the 0–20 Hz frequency band for NAc‐OB, NAc‐OFC, NAc‐VTA, and VTA‐OFC (a‐d), with minimal effects on VTA‐OB and OB‐OFC coherences (e‐f). Figure 7g displays the average coherence values within the 0–20 Hz for all LFP pairs across five mice (10 recordings), while Figure 7h illustrates the difference in coherence between MK‐801 and saline treatment. The results confirmed robust coherence between the NAc and VTA, as well as between the OB and OFC. Saline did not affect cross‐structure coherences, whereas MK‐801 notably increased LFP coherences between the NAc and other brain regions.

### 2.8 The impact of MK‐801 on SUA in the NAc During ODT

**Figure 8:**
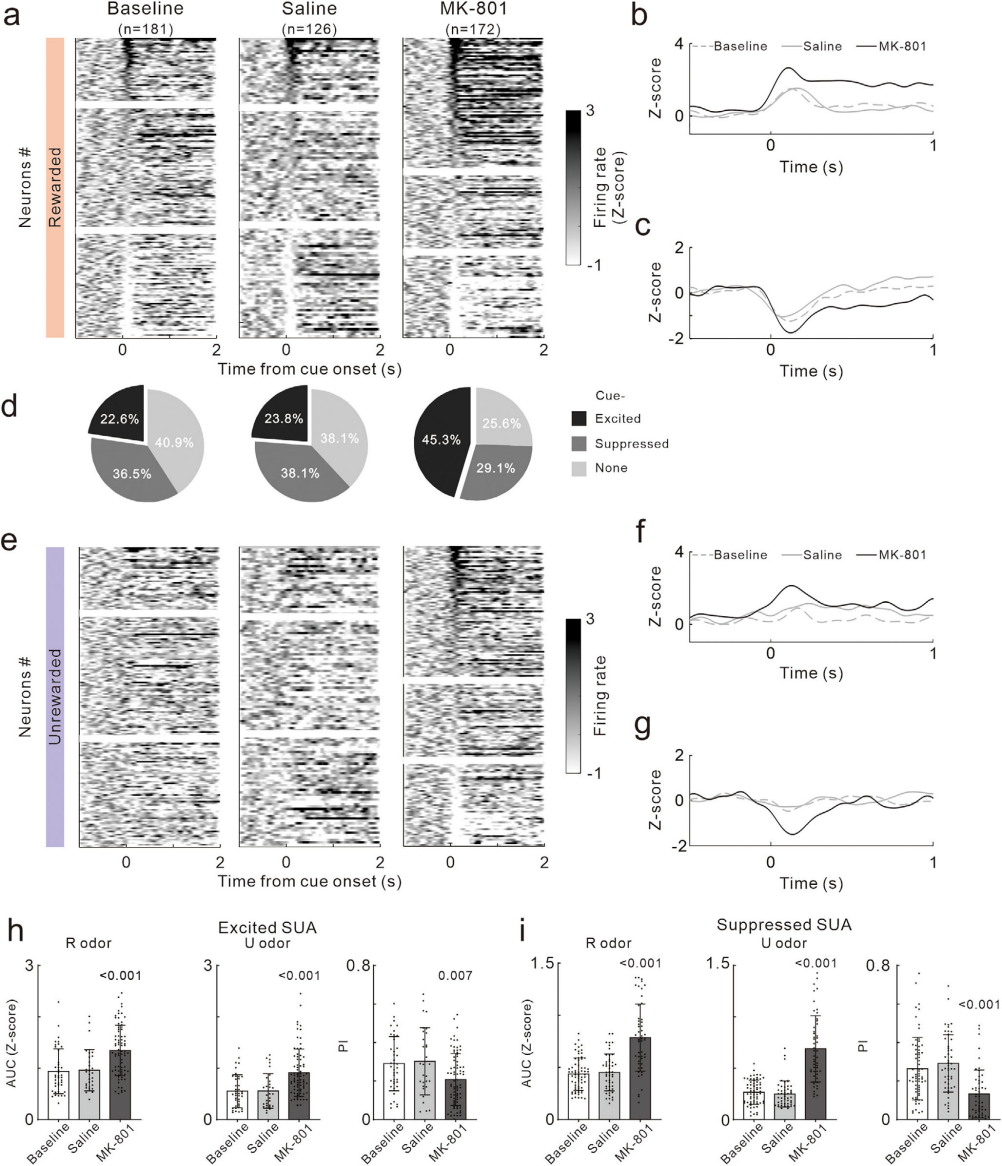
Effects of MK‐801 treatment on the SUAs of NAc in response to olfactory cues during ODT. a) Heat map showing PSTHs of all SUA recorded in 5 mice. Each row represents an individual PSTH averaged across all trials with rewarded odor cue. b,c) Mean PSTH for all SUAs classified as cue‐excited (b) and suppressed (c) under three conditions: baseline (gray dotted line), Saline (gray solid line), and MK‐801 (black solid line). d) Pie charts showing the proportion of cue‐excited, suppressed, or non‐responsive SUAs. e) Heat map showing PSTHs of all SUA in the trials with unrewarded odor cue. f,g) Mean PSTH for cue‐excited (f) and suppressed (g) SUAs. h) AUC of PSTH in the trials with rewarded (left) and unrewarded odor cue (middle) and DI (right) for cue‐excited SUAs. Dots represent individual value. Bars and ticks are means ± SD, one‐way ANOVA with Tukey's multiple comparisons test. i) Same as (h), but for cue‐suppressed SUAs.

Considering the pronounced increase in LFP theta oscillations in the NAc and coherences with other brain regions following MK‐801 treatment, we further detailed the SUA in the NAc during ODT using a 32‐channel electrode. **Figure** **8**a illustrates the Z‐scored PSTHs of all recorded SUAs, sorted by the difference in average firing rates within 1 s before and after the odor stimulus (arranged in descending order from top to bottom).

At baseline, the firing rates of some SUAs were increased, some were decreased and remaining SUAs were unaffected after odor R presented. The boundaries between these SUA types are marked by two white lines. At baseline, 22.6% of SUAs were excited units, 36.5% was suppressed units, and the remaining 40.9% was non‐responsive units. The average PSTHs of excited and suppressed SUAs are shown in Figure 8b,c, respectively. Following saline treatment, the proportion and average PSTH of the two SUA types remained unchanged (Figure 8d). However, after MK‐801 treatment, the proportion and firing rates of excited SUAs significantly increased, while those of suppressed unit significantly decreased (Figure 8a–d).

Figure 8e presents the responses of SUA to the odor U using the same sorting as in Figure 8a. At baseline, SUAs that exhibited excitatory or inhibitory responses to odor R showed diminished responses to odor U. This pattern remained unchanged following saline treatment. However, after MK‐801 treatment, both excited and suppressed SUAs also developed significant excitatory or inhibitory responses to the odor U (Figure 8e–g). In Figure 8h, we compared the AUC of the PSTHs for excited SUA among the Baseline, saline, and MK‐801 groups. The results indicated that the AUC for both odor R and U were significantly higher in the MK‐801 group compared to the Baseline and saline groups (F_(2146)_ = 13.92, *p <* 0.001; F_(2146)_ = 14.69, *p <* 0.001), with a corresponding decrease in DI (F_(2146)_ = 6.86, *p =* 0.001). Similarly, the AUC (negative) for suppressed SUA also increased significantly after MK‐801 treatment (F_(2161)_ = 40.54, *p <* 0.001; F_(2161)_ = 76.51, *p <* 0.001), with a significant decrease in DI (F_(2161)_ = 16.64, *p <* 0.001, Figure 8i). These findings suggested that MK‐801 enhanced the intensity of neuronal responses to odor stimulus in the NAc and reduced their selectivity.

**Figure 9:**
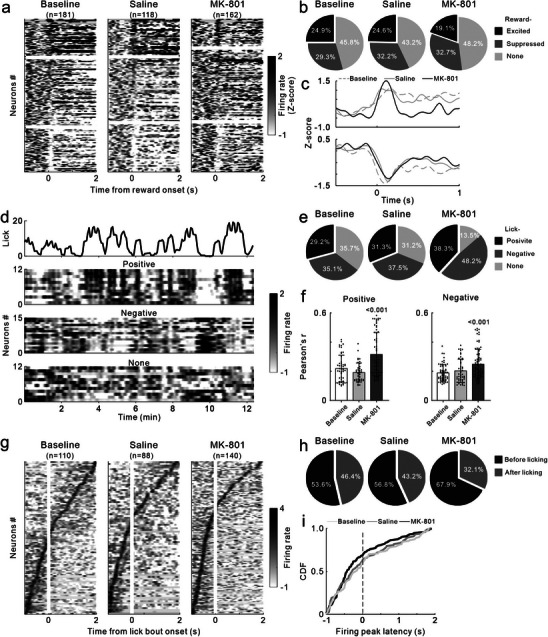
Effects of MK‐801 treatment on SUAs of NAc associating with reward and lick onset. a) Heat map showing PSTHs of all SUA relative to reward. b) Pie charts showing the proportion of reward‐excited, suppressed, or non‐responsive SUAs. c) Mean PSTH for reward‐excited and suppressed SUAs. d) Top: Time course of lick rate in one representative session. Bottom: PSTHs of SUA in the same session. Neurons exhibiting a positive correlation with lick rate are plotted in the upper part, those negatively correlated in the middle, and with no significant correlation in the lower part. e) Proportion of lick‐positive, negative, or non‐responsive SUAs. f) Pearson correlation coefficients of lick‐positive and negative SUAs. g) PSTHs of SUA relative to lick onset. h) Proportion of SUAs with a firing peak before and after licking. i) Cumulative distribution of firing peak latency relative to lick onset.

**Figure** **9**a displays the Z‐normalized PSTHs of SUA 1 s before and 2 s after reward delivery, sorted in descending order according to the response intensity evoked by the reward. At baseline, 24.9% of SUAs was classified as excited units, 29.3% as suppressed units, and the remaining 45.8% as non‐responsive units (Figure 9b). Following saline treatment, the distribution of SUA types remained consistent. However, MK‐801 treatment resulted in a decrease in the proportion of excited SUAs and an increase in non‐responsive SUAs. MK‐801 treatment also sharpened the excitatory response to reward, indicated by a higher response peak and a rapid decay of PSTH, and attenuated the suppressed response (Figure 9c). To investigate the relationship between neuronal activity and licking behavior, we computed Pearson correlation coefficients between the PSTHs of SUA and lick rate function. PSTHs were classified either as positively (top), negatively (middle), or not correlated (bottom) with licking activity (Figure 9d). Figure 9e illustrates the proportion of categorized PSTHs across the three experimental conditions. MK‐801 treatment increased the proportions of spike activities positively and negatively correlated to licking. Moreover, the coefficients of Pearson correlation were increased significantly by MK‐801 treatment (F_(2149)_ = 16.85, *p <* 0.001; F_(2183)_ = 9.472 *p <* 0.001, Figure 9f). We next examined neural activity related to lick bout onset (licks occurring >3 s after the previous lick) in NAc. All individual PSTH of SUA between 1 s before and 2s after lick bout onset were aligned according to their peak responses (Figure 9g). Under baseline conditions, 53.6% of SUAs peaked before the onset of licking (before licking), while the remaining 46.4% peaked afterward (after licking). saline treatment had no effect on the distribution of peak latencies of SUAs, whereas MK‐801 increased the proportion of SUA firing before licking (Figure 9h). The cumulative distribution functions (CDFs) of firing peak latency for SUAs confirmed that MK‐801 treatment significantly advanced the peak latency of neuronal firing relative to lick onset (Figure 9i).

### 2.9 The Therapeutic Effects of SEP on MK‐801‐Induced Response Inhibition Deficits, Abnormal DA Levels and SUA Responses in the NAc

**Figure 10:**
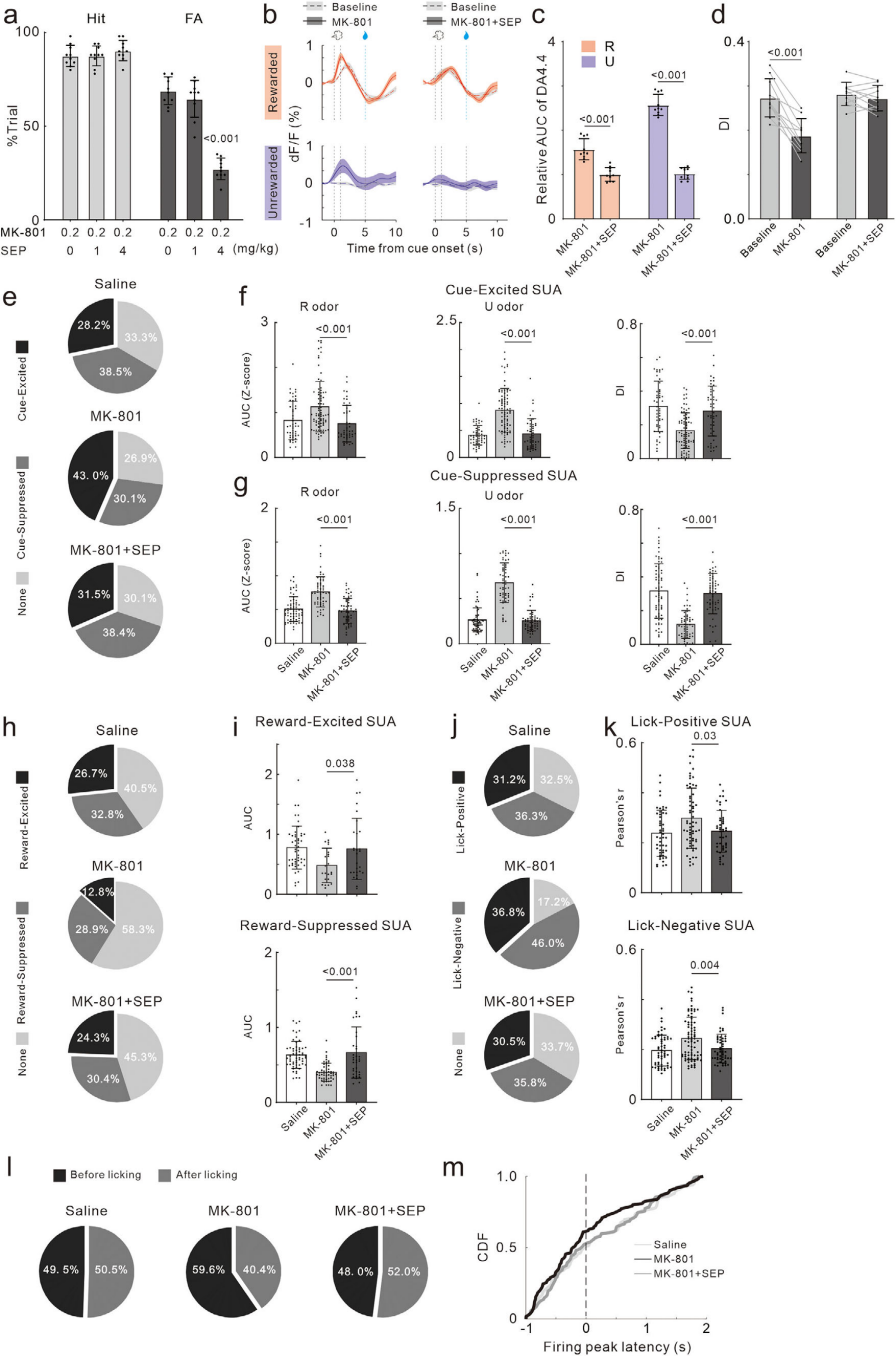
Ameliorative effects of SEP on MK‐801 induced abnormalities of behavior and neural activity. a) Statistical charts of Hit rate (light gray bars) and FA rate (dark gray bars) across different doses of SEP in combination with MK‐801. Dots represent the values of individual mouse. Bars and ticks are means ± SD from, one‐way ANOVA with Tukey's multiple comparisons test. b) Trial‐averaged traces of the DA signal responses evoked by Rewarded odor (top) and Unrewarded odor (bottom) in NAc. c) Relative AUC in R trials (red bars) and U trails (blue bars) for DA signal. Dots represent the values of individual mouse. Bars and ticks are means ± SD, Student's *t*‐test. d) Comparison of DI between baseline and post‐treatment in DA signal of two groups: MK‐801 and MK‐801+SEP treated. Paired Student's *t*‐test. e) Pie charts showing the proportion of cue‐excited, suppressed, or non‐responsive SUAs. f) AUC of PSTH in the trials with rewarded (left) and unrewarded odor cue (middle) and DI (right) for cue‐excited SUAs. One‐way ANOVA with Tukey's multiple comparisons test. g) Same as e, but for cue‐suppressed SUAs. h) Proportion of reward‐excited, suppressed, or non‐responsive SUAs. i) AUC of reward‐excited and reward‐suppressed SUAs. j) Proportion of lick‐positive, negative, or non‐responsive SUAs. k) Pearson correlation coefficients of lick‐positive and negative SUAs. l) Proportion of SUAs with a firing peak before and after licking. m) Cumulative distribution of firing peak latency relative to lick onset.

Based on our findings that MK‐801 elevated DA levels in the NAc, we investigated whether SEP, an agonist of the TAAR1, could correct the MK‐801‐induced deficits in response inhibition. After mice were well‐trained to discriminate between two odors, they were subjected to one of three treatments: 0.2 mg kg^−1^ MK‐801 + saline, MK‐801 + 1 mg kg^−1^ SEP, or MK‐801 + 4 mg kg^−1^ SEP. 1‐h post‐treatment, ODT was performed, with each experimental session using a different drug treatment and a 7‐day interval between sessions. Mice continued daily maintenance training between experiments, and the order of drug treatments was randomly assigned for each mouse. As shown in **Figure** **10**a, 4 mg kg^−1^ SEP effectively reduced MK‐801 induced the increase in FA rate (F_(2,27)_ = 85.46, *p <* 0.001). Furthermore, SEP restored MK‐801‐altered response patterns, including latency, frequency, and variance of licking. SEP also reversed MK‐801‐induced hyperlocomotion (Table S1, Supporting Information). SEP of 4 mg kg^−1^ had no significant effect on these performance measures in the non‐MK 801 treated mice (Table S2, Supporting Information). In addition, SEP reduced the increase of DA levels induced by MK‐801 in the NAc, as indicated by the DA 4.4 fluorescence (t_18_ = 6.33, *p <* 0.001; t_18_ = 18.03, *p <* 0.001, Figure 10b,c), and improved selectivity for odors R and U, as measured by the DI (t_9_ = 6.54, *p <* 0.001; t_9_ = 1.19, *p =* 0.27, Figure 10d). We further examined the impact of SEP on MK‐801‐induced abnormalities in SUA of NAc. Consistent with our previous results, MK‐801 increased the number of cue‐excited SUAs and decreased the number of cue‐suppressed SUAs (Figure 10e). And it also enhanced the response intensity of SUA to odor R and U (AUC, F_(2172)_ = 10.85, *p <* 0.001; F_(2172)_ = 42.03, *p <* 0.001, Figure 10f; F_(2176)_ = 35.69, *p <* 0.001; F_(2176)_ = 129.5, *p <* 0.001, Figure 10g) and reduced response selectivity (DI) (F_(2172)_ = 22.23, *p <* 0.001, Figure 10f; F_(2176)_ = 44.01, *p <* 0.001, Figure 10g). SEP treatment significantly corrected these cue‐related SUA abnormalities. The proportions of reward‐excited and reward‐suppressed SUA were both reduced after MK‐801 treatment (Figure 10h), and their response intensities to the reward were significantly diminished (F_(2,95)_ = 17.26, *p <* 0.001; F_(2134)_ = 43.82, *p <* 0.001, Figure 10i). But SEP treatment improved reward‐related SUA responses. Additionally, SEP reversed the MK‐801‐induced increase in the proportions of both lick‐positive and negative SUAs (Figure 10j) and reduced their correlation coefficients (Figure 10k). SEP also corrected the increase in the proportion of SUAs firing before licking and the advance in firing peak latency caused by MK‐801 (Figure 10l,m).

## 3 Discussion and Conclusion

In this study, we investigated for the first time the effects of the NMDAR antagonist (MK‐801) on impulsivity in mice and its underlying neural mechanisms. We found that MK‐801 increased the FA rate, shortened licking onset latency, elevated licking frequency and reduced inter‐trial consistency in mice performing ODT using a Go/No‐Go paradigm. Fiber photometry recordings revealed that neural calcium activities in the OFC, VTA and NAc were stronger in rewarded trials than in unrewarded trials in control mice, and MK‐801 reduced the difference. Typically, DA levels in the NAc, as measured by DA4.4, only transiently increased in response to rewarded cues. MK‐801 not only amplified DA release triggered by rewarded cues but also facilitated DA release in response to unrewarded cues. Electrophysiological recordings indicated that MK‐801 significantly enhanced theta oscillations in the NAc and its coherence with the OB, OFC, and VTA. Extracellular SUA recordings in the NAc showed that MK‐801 increased the response of SUA to cues but reduced the response selectivity for rewarded and unrewarded cues. Additionally, MK‐801 diminished the response evoked by reward, strengthened neural‐licking correlations, and advanced the firing peak latency relative to lick bout onset. Finally, we confirmed that SEP could correct MK‐801‐induced impulsive licking patterns, elevated DA release in the NAc, and abnormalities in SUA. These results reveal that MK‐801‐induced behavioral changes may correlate with an increase of DA release and abnormal neural excitability in the NAc. Furthermore, we also validated the therapeutic potential of SEP, offering new reference for the clinical treatment of impulsivity in SZ.

MK‐801, as a noncompetitive NMDAR antagonist, is widely utilized to establish SZ animal models by eliciting SZ‐like symptoms. In this study, we provided the first investigation of the aberrant performance in olfactory cued Go/No‐Go task in MK‐801‐treated mice. We found that MK‐801 induced less controlled licking pattern, as reflected by an increase in FA rate, premature responses, and high trial‐by‐trial variability. These findings are consistent with previous results characterizing impulsive behavior as often premature, poorly planned, and driven by reward‐seeking or immediate gratification without sufficient consideration of consequences.^[^ ^35^ ^]^ A noisy sensory processing in cortico‐thalamo‐cortical circuit had been well documented in SZ individuals and relevant rodent models,^[^ ^36^ ^]^ indicated by increased power of gamma oscillations.^[^ ^19^, ^37^ ^]^ The dysfunction of cortico‐thalamo‐cortical circuit may cause discrimination deficits. However, our results demonstrated that MK‐801 induced a significant elevation FA rate in ODT, whereas Hit rates remained unchanged. These effects are not attributable to a deficit in sensory discrimination, in that case, Hit and FA rates would be expected to increase similarly. We also found that systemic MK‐801 injection significantly increased total travel distance in OFT, indicating a generalized hyperactive or explorative state. Hyperactivity may facilitate premature licking by amplifying motor output and reducing behavioral restraint. Therefore, it is possible that MK‐801 increases impulsivity associated with a nonspecific enhancement of exploration and motor activity.

In fiber photometry recordings, we observed that calcium transient responses in the OB were triggered by both reward‐predictive and ‐unpredictive odors, showing no odor selectivity. Similar findings from previous OB fiber photometry studies have shown that calcium transient responses in OB neurons of naïve mice are independent of the reward significance of odors. Small differences in responses to reward‐predictive versus ‐unpredictive odors only emerge after extensive training, with the difference increasing to ≈1%.^[^ ^38^ ^]^ This contrasts with the OFC, which is often proposed to signal expected value, particularly for rewards.^[^ ^39^ ^]^ Previous experiments reported that cues predicting reward are generally reported to elicit stronger OFC neural activity than non‐reward‐predictive cues.^[^ ^40^, ^41^ ^]^ Consistent with this established role, our OFC recordings revealed that glutamatergic neurons exhibit significant selectivity for reward‐associated odors. It is well known that VTA neuron firing scales with the magnitude and unpredictability of received rewards.^[^ ^42^ ^]^ When the mice performed the conditioning task, VTA neurons exhibited a response to both reward‐predictive cues and unconditioned rewarding stimulation.^[^ ^43^, ^44^ ^]^ Our results indicated that calcium transient activity in VTA glutamatergic neurons exhibited peak responses only following reward‐predictive cues. This phenomenon may arise because, after training in the Go/No‐Go task, an association is established between the conditioned stimulus and the reward, thereby gradually reducing the unpredictability of received rewards. Consequently, glutamatergic neurons became more responsive to reward‐predictive cues and less reactive to expected rewards. In contrast, GABAergic neuronal activity showed a gradual increase before reward delivery, peaked after reward consumption, and remained elevated for an extended period. We hypothesize that the observed increase in GABAergic activity inhibits surrounding glutamatergic neurons, thereby attenuating their responsiveness to anticipated rewards. Additionally, GABAergic neurons in the VTA may regulate the precise timing of reward‐seeking behavior through the suppression of DA release. Under the regulation of the VTA, the activities of NAc neurons are also associated with cues and rewards.^[^ ^45^, ^46^ ^]^ Our results showed that NAc neural responses to reward‐predictive cues and reward were consistent with VTA. In summary, OB plays an important role in odor detection, and OFC involves in encoding the different olfactory sensations and the value of cues, while VTA and NAc process the information linking cue and reward.

Our study also revealed that MK‐801 treatment suppressed the transient response of GABAergic neurons in the OB, OFC and VTA, while enhancing the amplitude of responses in glutamatergic neurons without improving their response selectivity. Although MK‐801 did not abolish basic odor detection in the OB, it failed to enhance odor selectivity. In OFC and VTA, MK‐801 distorted reward valuation. Glutamatergic neurons previously unresponsive to the unrewarded odor became activated, while responses to the rewarded odor were not comparably enhanced. This asymmetrical pattern of neural activation indicates that MK‐801 altered licking patterns primarily by elevating the value or salience of unrewarded cues, leading to non‐selective licking responses to both odor stimuli.

In NAc, the vast majority of neurons, amounting to 99.7%, are GABAergic.^[^ ^32^ ^]^ MK‐801 treatment significantly enhanced the responses of GABAergic neurons but reduced the selectivity. Increased NAc neuronal activity is known to drive a general elevation in consummatory behavioral output,^[^ ^47^ ^]^ which manifested as a marked increase in lick rate to unrewarded cues, while having little impact on the already high lick rate to rewarded cues. Similar changes were observed in glutamatergic projections from VTA to NAc. Dynamics of DA level in NAc revealed that MK‐801 elevated DA release evoked by cues and reduced the cue selectivity. Excessive DA signaling enhanced overall GABAergic neuronal activity in the NAc, markedly adding the perceived value or salience of unrewarded stimuli, but exerting limited additional effects on the valuation of already learned rewarded stimuli.

It has been reported that MK‐801 preferentially targets and blocks NMDA receptors on GABAergic neurons.^[^ ^48^ ^]^ We hypothesized that MK‐801 diminished the activities of VTA GABAergic neurons by blocking glutamate binding to NMDARs, leading to disinhibition of DAergic neurons and enhanced DA release into the NAc.^[^ ^49^, ^50^ ^]^ This mechanism was proposed to intensify neural responses and reduce their selectivity in the NAc. These could be the primary cause of the altered response patterns in mice. In line with this, our in vivo electrophysiological recordings also revealed that MK‐801 predominantly enhanced LFP theta oscillations in the NAc. Theta oscillation is a type of brain wave pattern characterized by rhythmic neural activity in the frequency range of 4 to 8 Hz, which have recently received a considerable amount of attention and are considered essential to brain operations.^[^ ^51^ ^]^ In rodent studies, theta rhythms in the striatum are reported to involve in learning, decision‐making or timing.^[^ ^52^ ^]^ Our LFP recordings in the NAc during ODT demonstrated that theta power was significantly higher during FA trials compared to CR trials. The higher theta occurred at unrewarded cue presentation may trigger erroneous licking behavior. MK‐801 elevated theta power during both CR and FA trials, suggesting that NAc neurons exhibited enhanced value encoding of non‐rewarded cues. Our results also indicated that MK‐801 enhanced the LFP coherence between NAc and OB, OFC, and VTA. Synchronous activity within cortico‐striatal and VTA‐striatal has been associated with the processing of reward functions and motor behavior.^[^ ^53^, ^54^ ^]^ Moreover, extensive evidence has shown that theta oscillations in awake rodents are closely linked to locomotor states.^[^ ^55^ ^]^ Given the increase in NAc theta power during FA trials of the ODT, the MK‐801‐induced enhancement of NAc coherence with other regions may reflect a facilitation of the transformation from value/salience encoding to motor execution. Furthermore, through extracellular electrophysiological recordings, we found that the SUA of NAc had an obvious selectivity for cue, were modulated by reward, and showed a continuous contribution of firing peaks around lick onset. This is consistent with previously reported firing patterns of neurons in the striatum during Pavlovian conditioning tasks.^[^ ^56^ ^]^ MK‐801 treatment increased the reactivity of SUA to cues, reduced its modulation by the reward, strengthened its correlations with licking and shifted the response peak to precede the lick onset. These may be the neurophysiological basis underlying impulsivity in mice.

TAAR1 is a G‐protein‐coupled receptor expressed in cortical, limbic, and midbrain monoaminergic regions that modulate dopaminergic activity.^[^ ^57^ ^]^ SEP‐363856 (SEP), a TAAR1 agonist, has shown therapeutic effects on behavioral deficits in preclinical models of SZ and can normalize hyperdopaminergic activity.^[^ ^28^, ^58^ ^]^ Our results confirmed that SEP could correct MK‐801‐induced deficits, including elevated DA release, hyperexcitation of NAc neurons, and impaired response inhibition. SEP enhanced cue selectivity, restored reward preference, and decreased the probability of firing before lick onset, ultimately normalizing the response patterns in the Go/No‐Go task. These findings collectively suggest that SEP's therapeutic effects are mediated by its ability to normalize dopaminergic signaling and restore proper neuronal excitability and coding in the NAc, thereby improving the processing of value and the control of motor responses. Therefore, SEP has the potential to improve response inhibition and may offer a novel therapeutic option for the treatment of impulsivity in SZ patients.

### 3.1 Limitations

Although our study provides new insights into the pathophysiological mechanisms underlying impaired response inhibition induced by MK‐801, several limitations should be acknowledged.

First, acute systemic administration of MK‐801 does not fully capture the complex, chronic pathology of SZ. Specifically, it cannot recapitulate neurodevelopmental alterations, potential neurodegenerative processes, or sustained neurotransmitter dysregulation. Thus, the generalizability of our findings to chronic SZ remains limited. Moreover, systemic drug administration affects the entire brain, making it difficult to definitively attribute the observed effects solely to specific regions. Future studies should employ diverse models like chronic or genetic models to validate our findings. And it is required to utilize local microinjections or optogenetics to establish causal roles in target circuits.

Second, our methodological approach lacked the resolution to distinguish among distinct neuronal subpopulations within the VTA. We only used CaMKIIa‐GCaMP6 and mDlx‐GCaMP6 to monitor bulk activity in glutamatergic and GABAergic neurons, unable to identify DAergic neurons or those co‐releasing DA and glutamate, which involving in motor control.^[^ ^59^ ^]^ Furthermore, excessive phasic activity and dysfunctional neurotransmission of VTA DAergic neurons are well‐established in driving core aspects of SZ pathophysiology, particularly positive symptoms.^[^ ^60^, ^61^ ^]^ To overcome this limitation, future work should utilize more specific Cre‐dependent lines to clarify the contributions of different neuronal populations to the observed behavioral abnormalities.

Third, fiber photometry with the DA4.4 sensor enabled real‐time monitoring of dopamine dynamics during the ODT task with high temporal resolution. However, this method is an indirect measure of DA levels. Subsequent studies could employ microdialysis to provide a more comprehensive understanding of DA release and metabolism at the molecular level.

Forth, in addition to TAAR1 agonism, SEP is a partial agonist at the 5‐HT_1A_R. While our focus on TAAR1 aligns with its established role in modulating dopaminergic hyperactivity, a core feature of MK‐801‐induced deficits, we cannot exclude contributions from 5‐HT_1A_R agonism. 5‐HT_1A_R s are widely expressed in brain regions involved in mood, anxiety, and cognition,^[^ ^62^ ^]^ and their activation can modulate neuronal excitability and neurotransmitter release.^[^ ^63^ ^]^ These mechanisms may synergistically contribute to SEP's normalization of NAc hyperactivity and behavioral impulsivity. To resolve TAAR1 versus 5‐HT_1A_R mediation, co‐administration of SEP with selective antagonists (EPPTB for TAAR1, WAY‐100635 for 5‐HT_1A_R) will be helpful for building a comprehensive model of SEP's mechanism of action. Additionally, while this study focused on SEP‐363856′s therapeutic potential, future work should compare its efficacy against dopamine antagonists (e.g., BL‐1020^[^ ^64^ ^]^ and Lumateperone^[^ ^65^ ^]^) and newer muscarinic agonists (e.g., Xanomeline^[^ ^66^ ^]^) in reversing MK‐801‐induced deficits. Such comparative pharmacological studies would elucidate distinct neural mechanisms and inform new targeted strategies.

Finally, although our results demonstrate therapeutic effects of SEP on both MK‐801‐induced hyperactivity and impulsivity, it remains unclear whether these are causally related, and whether the therapeutic outcomes are depended on the effect of SEP on other brain regions beyond the VTA and NAc, such as the OFC.

## 4 Experimental Section

### Animals

C57BL/6 mice (Vital River laboratory, Beijing, China) were housed in group of 2–3 per cage with free access to food and water, and maintained on a 12:12 light/dark cycle. Both male and female mice were used in this study. The animals were maintained and treated in compliance with the policies and procedures detailed in the “Guide for the Care and Use of Laboratory Animals” of the National Institutes of Health. All experiment procedures were approved by the Animal Ethics Committee (CEUA) of China Medical University (No. KT2021031).

### Drugs Administration

MK‐801 (Sigma, St. Louis, MO, USA) and SEP‐363856 (SEP, MedChemExpress, Monmouth Junction, NJ, USA) were dissolved in 0.9% saline. Mice were administered i.p. injections of MK‐801 (0.2 mg kg^−1^) or SEP (2 or 4 mg kg^−1^).

### Open Field Test

The OFT was conducted in a 55 × 40 × 30 cm (length × width × height) arena constructed of non‐transparent black boards for well position tracking. Before testing, each mouse was brought into the procedure room singly and underwent 5 min of habituation in their home cage. Upon completion of the habituation period, the mouse was placed in the center of the open field arena. The total duration of OFT, excluding habituation, was 30 min.

### Go / No‐Go Tasks

Odors were delivered using a computer‐controlled olfactometer (RWD Life science, Shenzhen, China) that delivered a known concentration of an odor. Mice were deprived of water for 24 h to reach 80% of their baseline body weight and were maintained at this weight throughout the experiment.

Mice were initially habituated to be head‐fixed by just placing them repeatedly in the enclosure until they showed no obvious sign of discomfort. In all following phases, at the start of each session, the animal was head‐fixed and kept in the set‐up for 30 min without odor presentation to habituate to the set‐up and mitigate transport induced stress.

Behavioral testing was done in 3 phases: response training, rewarded cue training and Go / No‐Go task. In response training (≈2 days), the mice were initially trained to lick the reinforcement spout. Upon completing a sequence of three consecutive licks, they were given a water reward of 5 µL. Each mouse was required to receive a total of 1 mL of water in a single session. During rewarded cue training (≈3 days), the mice were presented with a 1s stimulus odor‐either rewarded (R, 1% isoamyl acetate, Sigma) or unrewarded (U, 1% phenyl acetate). When the mice were presented with the R, 10 µL of water reward was delivered 4s after the end of the stimulus, irrespective of their licking behavior at the spout. Then the mice were trained to perform the go / no‐go behavioral task. If the odor R was presented and the mouse responded by licking (Hit), a water reward (10 µL) was delivered through the spout 4s after the end of the stimulus. If the mouse failed to lick in response to the odor R (Miss), then the water reward was not delivered. When the odor U was delivered, no water reward was delivered regardless of the mouse's actions (if they licked, false alarm (FA); if did not lick, correct rejection (CR), see Figure 1). Thus, Hits and CRs were correct trials, and Misses and FAs were incorrect trials. Odors were presented following a pseudo‐randomized sequence, maintaining a 1:1 ratio. Each mouse completed 100 trials in a single session, with a 10 s interval between each trial. After 14 days of water‐restriction schedule, mice were given access to water ad libitum for at least two consecutive days. The Hit rate was calculated as the ratio of hit trials to go trials, and the FA rate was determined by the ratio of FA trials to no‐go trials. The formula of correct rate is as follows:

(1) $$Correctrate=Hitrate+\frac{1-FArate}{2}$$

Lick onset latency was defined as the first post‐cue time point at which the lick count exceeded a pre‐cue baseline threshold, which was calculated as the mean plus 1 standard deviation (SD) of licks during the 2‐s period before cue onset. Lick offset latency was defined as the last time point exceeding this same threshold. The CV was calculated as SD / average value.

### Virus Injection and Fiber Implantation

For fiber optic recordings, rAAV‐CaMKIIa‐GCaMP6‐WPRE‐hGH pA (PT‐0110, BrainVTA, Wuhan, China) or rAAV‐mDlx‐GCaMP6‐WPRE‐hGH pA (PT‐3025) was injected into unilateral OB (A*P =* +4.2, ML = +1.0, DV = −1.0 mm), OFC (A*P =* +2.8, ML = +0.5, DV = −2.5 mm), VTA (A*P =* −3.0, ML = +0.5, DV = −4.5 mm) or NAc (A*P =* +1.1, ML = +1.0, DV = −4.5 mm) to monitor neural activity. Additionally, to monitor DA level, rAAV‐hSyn‐DA4.4 (PT‐1340) was injected into unilateral NAc.

Mice were anesthetized with isoflurane (3 – 4% isoflurane for induction, 1 – 2% isoflurane for maintenance). Body temperature was maintained at ≈37 °C using a heating blanket. Eyes were covered with eye ointment to prevent drying. Subsequently, mice were fixed in a stereotaxic apparatus (#68 001, RWD Life science, Shenzhen, China). The skin was removed by a midline scalp incision and a small hole was drilled over the target site with a dental drill. AAVs were delivered unilaterally to the specified locations (≈0.3 µL per injection, ≈10 min per injection) at the corresponding depth beneath the skull surface using a beveled glass micropipette. After the injection, the pipette stayed in place for at least 5 min before it was withdrawn. Following the virus injection, an optical fiber (200 µm diameter; numerical aperture (NA) 0.37; NEWDOON, Hangzhou, China) was implanted at the injection site. The optical fiber was fixed in place with dental acrylic and an aluminum head plate was attached to the skull. Mice were individually housed for at least 10 days after surgery for recovery and to allow time for the expression of AAVs.

### Fiber Optic Recording

Mice initiated Go/No‐Go task training 2 weeks post the optical fiber implantation surgery. Once training was completed, fiber optic recordings were performed during task execution (consisting of 10 Go trials and 10 No‐Go trials in a pseudo‐randomized sequence, 10 s interval). A fiber optic cable was firmly attached to the implanted fiber optic cannula. Recordings were carried out by a fiber optic system (R811, RWD, Shenzhen, China). In brief, the 470 nm laser beams were used to excite the fluorescence emission of GCaMP6, DA4.4, 410 nm laser was used for motion control. The fluorescence signals were collected at 15 Hz.

### Surgical Procedures for In Vivo Electrophysiological Recording

Mice were anesthetized and fixed in a stereotaxic apparatus with the procedures similar above. The fur was removed with a fine trimmer. The skin was removed after disinfected with iodophor, then the skull was cleaned using a bone scraper. Two stainless screws were inserted into the cerebellum separately. The silver wires soldered to a pin connector were connected with stainless screws and served as ground and reference. Two metal headposts were attached to the skull with dental cement.

For LFP recordings at OB, OFC, VTA and NAc, a stainless‐steel guide tube (30G) was inserted into the brain at the targeting locations. After fixing the tube with dental cement, we lowered microwire electrodes (762 000, A‐M Systems, Hofheim, USA) into the tube and kept the tip at the level of 500 µm below the opening of the tube. Probes were coated with Dil‐dye (HY‐D0083, MedChemExpress) for post hoc recovery of the recording location (see below).

For SUA recordings at NAc, a 2 mm diameter cranial window was drilled with a 0.7 mm micro drill centered at the coordinates (A*P =* +1.1, ML = +1.0 mm). The cranial window is drilled down until a thin bone flap remains, which can be gently lifted away from the skull with forceps. After removal of dura, the 32‐channel electrode (NeuroNexus, USA) was clamped in an alligator clip attached to a stereotaxic arm to position the electrode tips above the craniotomy and slowly inserted into the brain. Wires were lowered in areas with as few visible blood vessels as possible to avoid hemorrhage during implantation. Drops of saline were used to fill the cranial window, and a thin layer of superglue was applied to the skull surface to seal the craniotomy. Dental cement was then applied over the superglue layer to further isolate the external environment from the cranial window. The implant was secured to anchor screws and the remaining exposed wires covered in dental cement. After recovery from anesthesia, mice were individually housed for at least 10 days.

### LFP Recording

After recovery from the surgery of microwire electrodes implantation, LFP recordings were conducted in an electrically shielded, sound‐proof box. The mice were accustomed to head fixation and familiarized with the environment for 30 min where they were affixed atop a free‐spinning treadmill through the custom headposts on the skull. The mouse could move its body while the head was fixed. The recording experiments were performed on the fifth day. Raw signals were digitized with a multichannel extracellular amplifier (RA16PA; Tucker‐Davis Technologies, TDT; Alachua, FL, USA). And they were band‐pass filtered to extract LFP (1–300 Hz), then imported into computer for further analysis.

### SUA Recording

After 1 week recovery from the surgery of electrodes implantation, mice initiated Go/No‐Go task training. SUA recordings were conducted during task execution (consisting of 25 Go trials and 25 No‐Go trials in a pseudo‐randomized sequence, 10 s interval) in an electrically shielded, sound‐proof box. Raw signals were digitized with a multichannel extracellular amplifier (RA16PA; Tucker‐Davis Technologies, TDT; Alachua, FL, USA). And they were band‐pass filtered to extract spike activity (300–5000 Hz), then imported into computer for further analysis. SUA was isolated using a wavelet‐based spike sorting package (OpenSorter software, TDT). We considered 3 SDs above baseline as the threshold to distinguish spikes. K‐means clustering method in OpenSorter was adopted to sort single‐unit spikes. A unit with the largest amplitude and normal overlaid spike profile was chosen from each electrode. Another criterion was that the number of spikes with an interspike interval of <2 ms in the histogram should be <0.2% of the total number of spikes. Subsequent analysis included only well‐isolated units.

### Analysis for Fiber Optic Recording

Data were segmented according to the onset of odor stimulation on individual trials. This work calculated the optical signal value F as F470/F410 and then determined dF/F = (F –F0) / F0, where F0 is the baseline fluorescent signal averaged over a 2 s long control time window, which preceded the onset of odor stimulation. The dF/F values were presented as average plots with a shaded area indicating the standard error (SE). Area under the curve (AUC) was calculated from 1 s prior to 10 s post initiation of odor stimulus onset. We then calculated the relative AUC and discrimination index (DI) to evaluate the impact of drug injections on the neural responses to odor R and U. The formulas are as follows:

(2) $$Relative\;AUC=\frac{AUC_{post}}{AUC_{baseline}}$$

(3) $$DI=\frac{\left|AUC_{R}-AUC_{U}\right|}{AUC_{R}+AUC_{U}}$$

### Analysis for LFP Recording

The power spectrogram and cross‐structure coherence of LFP signals were analyzed using the “mtspecgramc” and “cohgramc” functions from the Chronux toolbox, respectively, as implemented in custom‐written code (http://chronux.org/).^[^ ^67^ ^]^ The parameters for function used were: movingwin = [5 1] or [10 1], sampling frequency (fs) = 1000, zero‐padding (pad) = 0, f pass = [0 20], tapers = [3 5], nfft = 8192, noverlap *=* 80%. The trial‐based spectra of LFPs were analyzed using a wavelet‐based analysis algorithm, implemented in custom‐written code using the eeglab toolbox (https://sccn.ucsd.edu/eeglab/index.php).

### Analysis for SUA Recording

For visualizing neural activity, this work computed Z‐scored peristimulus time histograms (PSTHs) using non‐overlapping bins of 5 ms, smoothed with a Gaussian Kernel (width = 50 ms). For the analysis of SUA, trial‐averaged PSTHs within different temporal windows (from 1 s prior to 2 s post initiation of cue, reward or lick onset) were computed and presented as heatmap. SUAs were classified as cue‐excited if they exceeded the 99.9% confidence interval of a Poisson distribution comprised of a 10 s pre‐cue baseline for at least one 50 ms bin during the time window of 0–500 ms post‐cue onset. SUAs were classified as cue‐suppressed if they fell below the 99.9% confidence interval for at least one 50 ms bin. The remaining SUAs were categorized as non‐responsive units. Similarly, SUAs were classified as significantly reward‐excited or suppressed if firing exceeded or fell below the threshold for at least one 50 ms bin during the time window of 0–500 ms post‐reward onset. To quantify cue‐ and reward‐related modulation, we calculated the AUC and DI of PSTH.

To analyze correlation between SUA and licking, the Pearson's correlation coefficient between each PSTH of SUA and lick rate function was calculated. This work used a circular shift permutation test to randomize lick rate values while preserving their temporal structure. The Pearson's correlation coefficient was then recalculated between the time‐shifted lick rate and the original PSTH of SUA. This helped to compare the Pearson's coefficient of each neuron against its distribution of shifted correlation coefficients. The neuron was considered significantly positive if the coefficient was higher than 95% of all time‐shifted correlation coefficients. A neuron was negative if the coefficient was lower than 95% of shuffled coefficients, and all other neurons were not significant. Additionally, this work classified the SUAs into two categories based on the timing of the peak in each PSTH: “before licking” (occurring from −1 to 0 s relative to the onset of the lick bout) and “after licking” (from 0 to 2 s).

### Immunofluorescence

At the end of the experiments, this work verified the recording electrode locations and the expression of GCaMP6 and DA4.4 to confirm their restriction to the target brain regions. Mice were anesthetized with pentobarbital (100 mg kg^−1^) and perfused with ≈10 mL phosphate‐buffered saline (PBS) followed by ≈20 mL 4% paraformaldehyde in PBS. The brains were removed and immersed in 30% sucrose solution overnight before being sectioned at 20 µm thickness via a freezing microtome (FS800, RWD, Shenzhen, China). The brain slides were washed and mounted in an anti‐fade reagent with DAPI (SL1841, Coolaber, 562 Beijing, China).

### Statistical Analysis

Data were compared via *t*‐tests for comparisons between two groups and one‐way ANOVA with Turkey's post hoc test for comparisons among multiple groups. Values in the text are reported as mean ± SD unless reported otherwise. Results were considered statistically significant when the *p* value < 0.05. Custom‐written Python programs using numpy and scipy were used for our statistical analysis.

## Conflict of Interest

The authors declare no conflict of interest.

## Author Contributions

Conceptualization: L.Q., P.Y., Methodology: X.W., Q.L., X.W., Z.L., Investigation: X.W., Q.L., H.Y., Visualization: X.W., Supervision: L.Q., P.Y., Writing—original draft: X.W., Writing—review & editing: L.Q., P.Y.

## Supporting information



Supporting Information

## Data Availability

The data that support the findings of this study are available from the corresponding author upon reasonable request.
