# Supplementary material for: Cortico‐Striatal‐Midbrain Circuit Dysregulation Underlying MK‐801 Induced Impulsivity and the Ameliorative Effects of SEP
Source: Adv Sci (Weinh). 2025 Oct 24;13(3):e02079. doi: 10.1002/advs.202502079 (PMC12806448; doi:10.1002/advs.202502079)
Supplement: Supplementary file 1 — Supporting Information [file ADVS-13-e02079-s001.docx]

**Figure S1.** **Effects of MK-801 treatment on behaviors and LFP theta power.**


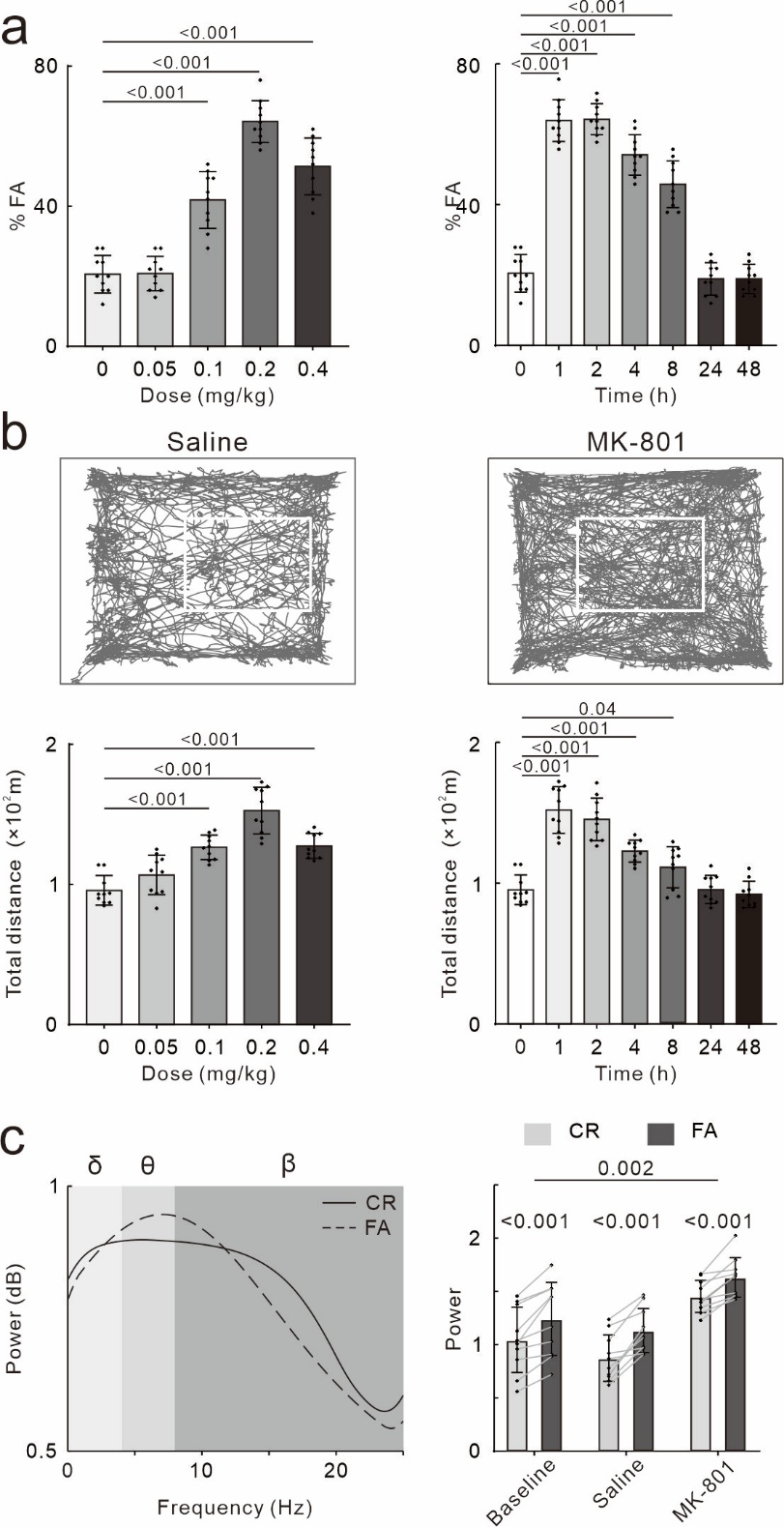


(a) FA rate under different doses of MK-801 (left) and at various time points following injection of 0.2 mg/kg MK-801 (right). (b) Top, Movement trace diagrams of the Saline and MK-801-treated mice in the OFT (the white box indicates the central area). Bottom, total distance traveled under different doses of MK-801 (left) and at various time points following injection of 0.2 mg/kg MK-801 (right). (c) Representative LFP power spectra from CR (black line) and FA (dashed line) trials at baseline. Gray shadings indicate the frequency bands of delta, theta and beta. (d) Statistical charts of the power of theta band. Dots represent the values of individual mouse. Bars and ticks are means ± SD, Two-way ANOVA with Šídák's post-hoc test.

**Table S1 Ameliorative effects of SEP on MK-801 induced abnormalities of behavior.**

|  | MK-801 | MK-801+SEP (1mg/kg) | MK-801+SEP (4 mg/kg) | P |
| --- | --- | --- | --- | --- |
| Onset latency (s) | 1.76±0.07 | 1.79±0.05 | 2.28±0.10 | <0.001 |
| CV of onset latency | 0.46±0.07 | 0.44±0.07 | 0.24±0.03 | <0.001 |
| Offset latency (s) | 7.79±0.44 | 7.80±0.10 | 7.86±0.14 | 0.82 |
| CV of offset latency | 0.16±0.02 | 0.16±0.01 | 0.11±0.01 | <0.001 |
| Lick frequency (Hz) | 3.39±0.33 | 3.53±0.27 | 2.85±0.16 | <0.001 |
| CV of lick frequency | 0.34±0.03 | 0.37±0.05 | 0.31±0.04 | 0.005 |
| Total distance (×10^2^ m) | 1.61±0.20 | 1.50±0.15 | 0.88±0.28 | <0.001 |

**Table S2 Effects of SEP treatment on behaviors.**

|  | Saline | SEP  (1 mg/kg) | SEP  (4 mg/kg) | P |
| --- | --- | --- | --- | --- |
| Hit rate (%) | 90.20±5.20 | 85.00±5.60 | 87.00±5.44 | 0.11 |
| FA rate (%) | 24.80±4.44 | 20.60±5.66 | 20.20±5.03 | 0.10 |
| Onset latency (s) | 2.48±0.07 | 2.50±0.05 | 2.47±0.04 | 0.68 |
| CV of onset latency | 0.19±0.03 | 0.18±0.01 | 0.19±0.01 | 0.76 |
| Offset latency (s) | 7.70±0.24 | 7.79±0.15 | 7.75±0.20 | 0.61 |
| CV of offset latency | 0.11±0.02 | 0.10±0.01 | 0.10±0.01 | 0.08 |
| Lick frequency (Hz) | 2.67±0.23 | 2.75±0.22 | 2.64±2.22 | 0.55 |
| CV of lick frequency | 0.29±0.05 | 0.28±0.04 | 0.26±0.03 | 0.14 |
| Total distance (×10^2^ m) | 0.99±0.20 | 0.97±0.25 | 0.89±0.31 | 0.65 |
